# The Regulatory Status Adopted by Lymph Node Dendritic Cells and T Cells During Healthy Aging Is Maintained During Cancer and May Contribute to Reduced Responses to Immunotherapy

**DOI:** 10.3389/fmed.2018.00337

**Published:** 2018-11-30

**Authors:** Joanne K. Gardner, Connie Jackaman, Cyril D. S. Mamotte, Delia J. Nelson

**Affiliations:** ^1^School of Pharmacy and Biomedical Sciences, Faculty of Health Sciences, Curtin University, Perth, WA, Australia; ^2^Curtin Health and Innovation Research Institute, Curtin University, Perth, WA, Australia

**Keywords:** dendritic cells, aging, cancer, immunotherapy, immunosuppression

## Abstract

Aging is associated with an increased incidence of cancer. One contributing factor could be modulation of immune cells responsible for anti-tumor responses, such as dendritic cells (DCs) and T cells. These immunological changes may also impact the efficacy of cancer immunotherapies in the elderly. The effects of healthy aging on DCs and T cells, and their impact on anti-mesothelioma immune responses, had not been reported. This study examined DCs and T cells in young (2–5 months; equivalent to 16–26 human years) and elderly (20–24 months; equivalent to 60–70 human years) healthy and mesothelioma-bearing C57BL/6J mice. During healthy aging, elderly lymph nodes adopted a regulatory profile, characterized by: (i) increased plasmacytoid DCs, (ii) increased expression of the adenosine-producing enzyme CD73 on CD11c^+^ cells, and (iii) increased expression of multiple regulatory markers (including CD73, the adenosine A2B receptor, CTLA-4, PD-1, ICOS, LAG-3, and IL-10) on CD8^+^ and CD4^+^ T cells, compared to lymph nodes from young mice. Although mesotheliomas grew faster in elderly mice, the increased regulatory status observed in healthy elderly lymph node DCs and T cells was not further exacerbated. However, elderly tumor-bearing mice demonstrated reduced MHC-I, MHC-II and CD80 on CD11c^+^ cells, and decreased IFN-γ by CD8^+^ and CD4^+^ T cells within tumors, compared to young counterparts, implying loss of function. An agonist CD40 antibody based immunotherapy was less efficient at promoting tumor regression in elderly mice, which may be due to: (i) failure of elderly CD8^+^ T cells to up-regulate perforin, and (ii) increased expression of multiple regulatory markers on CD11c^+^ cells and T cells in elderly tumor-draining lymph nodes (including CD73, PD-1, ICOS, LAG-3, and TGF-β). Our findings suggest that checkpoint blockade may improve responses to immunotherapy in elderly hosts with mesothelioma, and warrants further investigation.

## Introduction

There is an increased incidence of cancer with aging, and one contributing factor could be age-induced modulation of immune cells that mediate anti-tumor immune responses, such as dendritic cells (DCs) and T cells. DCs are antigen-presenting cells that play an important role in initiating anti-tumor immune responses, on account of their ability to activate tumor-specific CD8^+^ cytotoxic T cells, which can directly kill tumor cells and mediate tumor regression (1–5). There is some evidence that aging influences DC anti-tumor function, with DCs from elderly mice shown to have a reduced ability to stimulate tumor-specific cytotoxic CD8^+^ T cells *in vivo* (6, 7). Furthermore, administration of DC vaccines to elderly tumor-bearing mice leads to generation of weak cytotoxic T cell activity, and does not slow tumor growth, resulting in a shorter survival time (8, 9). Age-related defects in murine T cell anti-tumor function have also been reported, these include; reduced numbers of tumor-antigen-specific T cells, decreased proliferative capacity, impaired cytotoxic activity, and reduced production of effector cytokines, such as interferon (IFN)-γ and IL-2, in elderly tumor-bearing mice (10–18). However, the effects of healthy aging on DCs and T cells, and the potential impact on generation of anti-tumor immune responses in mesothelioma, an asbestos-induced cancer which occurs predominantly in elderly populations aged 60 years and above (19, 20), have not yet been reported.

Furthermore, age-related changes in DCs and T cells may impact on the efficacy of cancer immunotherapies in the elderly. The few studies performed to-date that have considered aging indicate that cancer immunotherapies are less effective in elderly hosts (6, 8, 9, 11, 21–25). Little is known about the effects of aging on responses to immunotherapy in mesothelioma. Our previous studies, using young mice (1.5–2 months of age, equivalent to 16–26 human years), have shown that intra-tumoral administration of IL-2 in combination with agonist anti-CD40 antibody (IL-2/CD40) induces permanent regression of large AE17 mesothelioma tumors mediated by CD8^+^ T cells, neutrophils (26), B cells (27) and pro-inflammatory M1 macrophages (28). Cured mice remained tumor-free for the remainder of their natural lives and were protected from tumor re-challenge by CD8^+^ and CD4^+^ T cells and natural killer cells (29, 30). Studies from our laboratory have also shown that elderly macrophages activated with *in vitro* IL-2 and agonist anti-CD40 antibody restore the capacity of elderly CD8^+^ T cells to produce IFN-γ and perforin (31, 32). Here, we extend these studies to investigate the influence of aging on DC and T cell function during treatment with IL-2/CD40 *in vivo*.

The aim of this study was to examine the influence of aging on DC and T cell function in healthy mice vs. mice with mesothelioma, and to examine responses to IL-2/CD40 immunotherapy in elderly and young mesothelioma-bearing mice. We compared DCs and T cells from young (2–5 months; equivalent to 16–26 human years) (33) and elderly C57BL/6J mice (20–24 months; equivalent to 60–70 human years) (33, 34) in: (i) lymph nodes of healthy mice, and (ii) tumor-draining lymph nodes and tumors of mesothelioma-bearing mice, with and without IL-2/CD40 immunotherapy. We examined the effects of aging on DC and T cell subset proportions, as well as expression of activation/effector and regulatory markers.

## Materials and methods

### Mice

This project was approved by the Curtin University Animal Ethics Committee (approval number AEC-2012-21), and all experiments were performed according to the Australian Code of Practice for the care and use of animals for scientific purposes. Young (2–5 months; equivalent to 16–26 human years) (33) and elderly (20–24 months; equivalent to 60–70 human years) (33, 34) female C57BL/6J mice were obtained from the Animal Resources Center (Western Australia, Australia) and maintained under specific pathogen-free conditions in the Curtin University animal facility. For studies involving healthy mice, any mice that had a palpable mass, enlarged lymph nodes, enlarged spleen, or enlarged liver were excluded to ensure that only healthy, non-tumor-bearing mice were examined.

### Murine mesothelioma cell line culture

AE17 is a murine mesothelioma cell line derived from the peritoneal cavity of C57BL/6J mice injected with asbestos fibers, as previously described (35). AE17sOVA was developed by transfecting the AE17 parental cell line with cDNA coding for secretory ovalbumin (OVA), hence ovalbumin, which contains the SIINFEKL peptide, functions as a marker tumor antigen, as previously described (35). AE17 cells were cultured in complete medium, consisting of RPMI 1640 media (Invitrogen, California, USA), supplemented with 10% fetal calf serum (FCS; ThermoScientific, Victoria, Australia), 100 units/ml of penicillin and 100 μg/ml streptomycin (Penicillin-Streptomycin; Life Technologies, Victoria, Australia) and 2 mM L-glutamax (Life Technologies). AE17sOVA cells were maintained in complete medium supplemented with 400 μg/L of the neomycin analog G418 (Geneticin; Life Technologies, Grand Island, USA). All cells were cultured at 37°C in a 5% CO_2_ atmosphere.

### Murine tumor growth and treatment with IL-2/CD40 immunotherapy

For tumor inoculation, mice were injected subcutaneously in the right flank with 5 x 10^5^ AE17 or AE17sOVA tumor cells per mouse, in 100 μl of PBS. Tumor growth was monitored and tumor size was measured daily using microcallipers. The maximum tumor size allowed was 140 mm^2^ in accordance with ethics approval from the Curtin University Animal Ethics Committee.

For immunotherapy, AE17 or AE17sOVA tumor-bearing mice were treated intra-tumorally once every 2-3 days with PBS diluent (100 μl/dose) or interleukin-2 (IL-2, 20 μg/dose; Cetus Corporation, California, USA) and agonist anti-CD40 antibody (FGK45, 40 μg/dose; Absolutions, Western Australia, Australia) as previously described (26), for 5 doses in total.

### Processing and staining murine tissues for flow cytometric analysis

Mice were euthanized using methoxyflurane (Medical Developments International, Victoria, Australia). Spleens and lymph nodes were collected from healthy (non-tumor-bearing) mice, and tumors and tumor-draining lymph nodes were collected from AE17 tumor-bearing mice for flow cytometric analysis. For samples that required intracellular cytokine staining, murine tissues were collected into FACS buffer [1 × PBS/1% newborn calf serum (NCS)/1% bovine serum albumin (BSA)] + 2.5 μg/ml brefeldin A (Sigma), and incubated for 4 h prior to staining. Lymph nodes and tumors were disaggregated into single-cell suspensions by gentle dispersion between two frosted glass slides.

Single-cell suspensions of murine lymph nodes and tumors were stained with fluorescently-labeled antibodies to identify DC subsets (CD11c, CD11b, CD8, CD4, B220, and GR-1; Table 1), T cell subsets (CD3, CD8, CD4, CD25, and FoxP3; Table 1), and activation (MHC-I, MHC-II, CD40, CD80, CD86, IFN-γ, IL-12, TNF-α, and/or perforin; Table 1) and regulatory markers (CD39, CD73, A2AR, A2BR, PD-L1, GAL-9, IL-10, TGF-β latency-associated peptide, ICOS, LAG-3, CTLA-4, and/or PD-1; Table 1).

**Table 1:** Anti-mouse antibodies used in this study.

**Antigen**	**Conjugate**	**Clone**	**Supplier**
**SURFACE ANTIGENS/MARKERS**			
Adenosine A2B receptor	Purified	Polyclonal	Alomone Labs
B220 (CD45R)	Biotin	RA3-6B2	Biolegend
CD3e	APC-eFluor780	17A2	eBioscience
CD3e	PE	145-2C11	BD
CD4	APC-Cy7	GK1.5	Biolegend
CD4	PerCP-Cy5.5	RM4-5	BD
CD8α	AF405	5H10	Invitrogen
CD8α	AF647	5H10	Invitrogen
CD8α	FITC	53-6.7	Biolegend
CD8α	PerCP-Cy5.5	53-6.7	BD
CD11b	PE-Cy7	M1/70	BD
CD11c	APC	HL3	BD
CD11c	APC	N418	Invitrogen
CD11c	Biotin	N418	Biolegend
CD11c	FITC	HL3	BD
CD25	PE-Cy7	PC61	Biolegend
CD39	Biotin	5F2	Biolegend
CD40	PE	3/23	BD
CD73	PE	TY/11.8	Biolegend
CD73	PerCP-Cy5.5	TY/11.8	Biolegend
CD80	BV421	16-10A1	BD
CD86	FITC	GL1	Biolegend
GR-1 (Ly6C/Ly6G)	PE	RB6-8C5	BD
ICOS (CD278)	Pacific Blue	C398.4A	Biolegend
LAG-3 (CD223)	PerCP-Cy5.5	C9B7W	Biolegend
MHC-I (H-2Kb)	PerCP-Cy5.5	AF6-88.5	Biolegend
MHC-II (I-A/I-E)	APC-Cy7	M5/114.15.2	Biolegend
PD-1 (CD279)	Biotin	RMP1-30	Biolegend
PD-L1 (CD274)	PE-Cy7	10F.9G2	Biolegend
**INTRACELLULAR ANTIGENS/MARKERS**			
Adenosine A2A receptor	PerCP-Cy5.5	7F6-G5-A2	Santa Cruz
CTLA-4 (CD152)	BV421	UC10-4B9	Biolegend
FoxP3	AF647	150D	Biolegend
Galectin-9	PerCP-Cy5.5	RG9-35	Biolegend
IFN-γ	PE-Cy7	XMG1.2	Biolegend
IL-10	BV421	JES5-16E3	Biolegend
IL-12 (p40/p70)	PE	C15.6	BD
Perforin	APC	eBio0MAK-D	eBioscience
TGF-β1 latency-associated peptide	Biotin	TW7-16B4	Biolegend
TNF-α	AF647	MP6-XT22	Biolegend

Samples were incubated with surface primary antibodies (diluted in FACS buffer + 2.5 μg/ml brefeldin A) for 30 min at 4°C in the dark, and washed twice in FACS buffer + 2.5 μg/ml brefeldin A by centrifuging at 1,200 rpm for 2 min. Samples stained with purified A2BR antibody or biotinylated primary antibodies (B220, CD11c, CD39, and PD-1) were incubated with goat anti-rabbit IgG-AF488 (Invitrogen) or streptavidin-V500 (BD), respectively (diluted in FACS buffer + 2.5 μg/ml brefeldin A), for 30 min at 4°C in the dark. Samples were washed twice in FACS buffer + 2.5 μg/ml brefeldin A, followed by two PBS washes, then stained with Zombie green or Zombie NIR viability dyes (both from Biolegend) for 15 min at 4°C in the dark, washed twice in FACS buffer, then twice in PBS. Cells were fixed in 1% paraformaldehyde (Sigma) diluted in PBS by incubating for 20 min at 4°C in the dark, washed twice with FACS buffer, and permeabilised with FACS buffer + 0.1% saponin (Sigma) for 15 min at 4°C in the dark. For samples requiring FoxP3 staining, cells were fixed using FoxP3 Fix/Perm buffer (Biolegend), permeabilised using FoxP3 Perm buffer (Biolegend), then stained with FoxP3 antibody (1 μl/well) for 30 min at 4°C in the dark, washed twice in FACS buffer + 0.1% saponin, then stained with other intracellular antibodies. Cells were stained with intracellular primary antibodies (diluted in FACS buffer + 0.1% saponin) for 30 min at 4°C in the dark, then washed twice in FACS buffer. Samples stained with TGF-β-biotin were incubated with streptavidin-V500 (diluted in FACS buffer + 0.1% saponin) for 30 min at 4°C in the dark, followed by two washes using FACS buffer, and resuspended in 200 μl of FACS buffer per well. Samples were stored at 4°C in the dark (up to a maximum of 1 week) before analysis on a FACSCanto II using FACSDiva software (BD) or FlowJo software (TreeStar, USA).

### *In vivo* cytotoxic T lymphocyte (CTL) assay for analysis of CTL function

The cytotoxic activity of tumor-specific CD8^+^ T cells was assessed via an *in vivo* CTL assay, as previously described (27). Briefly, target cells for this assay were derived from spleen and lymph node cells from healthy young C57BL/6J mice. Spleen and lymph node cell suspensions were RBC-lysed, washed and divided into two populations. One population was pulsed with 10^−6^ M SIINFEKL peptide for 90 min at 37°C, washed with PBS, and labeled with a high concentration (5 μm) of carboxyfluorescein succinimidyl ester (CFSE; Molecular Probes, Oregon, USA). Control target cells (i.e., not pulsed with peptide) were labeled with a low concentration of CFSE (0.5 μm). 10^7^ cells from each population were pooled in 200 μl PBS and intravenously injected into each recipient AE17sOVA-bearing young or elderly mouse. Tumor-draining lymph nodes and tumors were collected from young and elderly recipient mice 24 h after target cell injection, and the number of cells in each target cell population in each tissue measured by flow cytometry. The ratio between the percentages of unpulsed vs. SIINFEKL-pulsed cells (CFSE^lo^/CFSE^high^) was calculated to obtain a numerical value of cytotoxicity. To normalize data, allowing inter-experimental comparisons, ratios between the percentages of peptide-pulsed cells in control (tumor-free mice and tumor-bearing PBS-treated mice) vs. age-matched tumor-bearing mice were calculated.

### Data analysis

Statistical differences between young and elderly populations were calculated using a Mann-Whitney *U*-test, using GraphPad PRISM v7 (GraphPad Software Inc, USA). *P* < 0.05 were considered statistically significant.

## Results

### AE17 mesothelioma tumors grow faster in elderly mice

**Figure 1:**
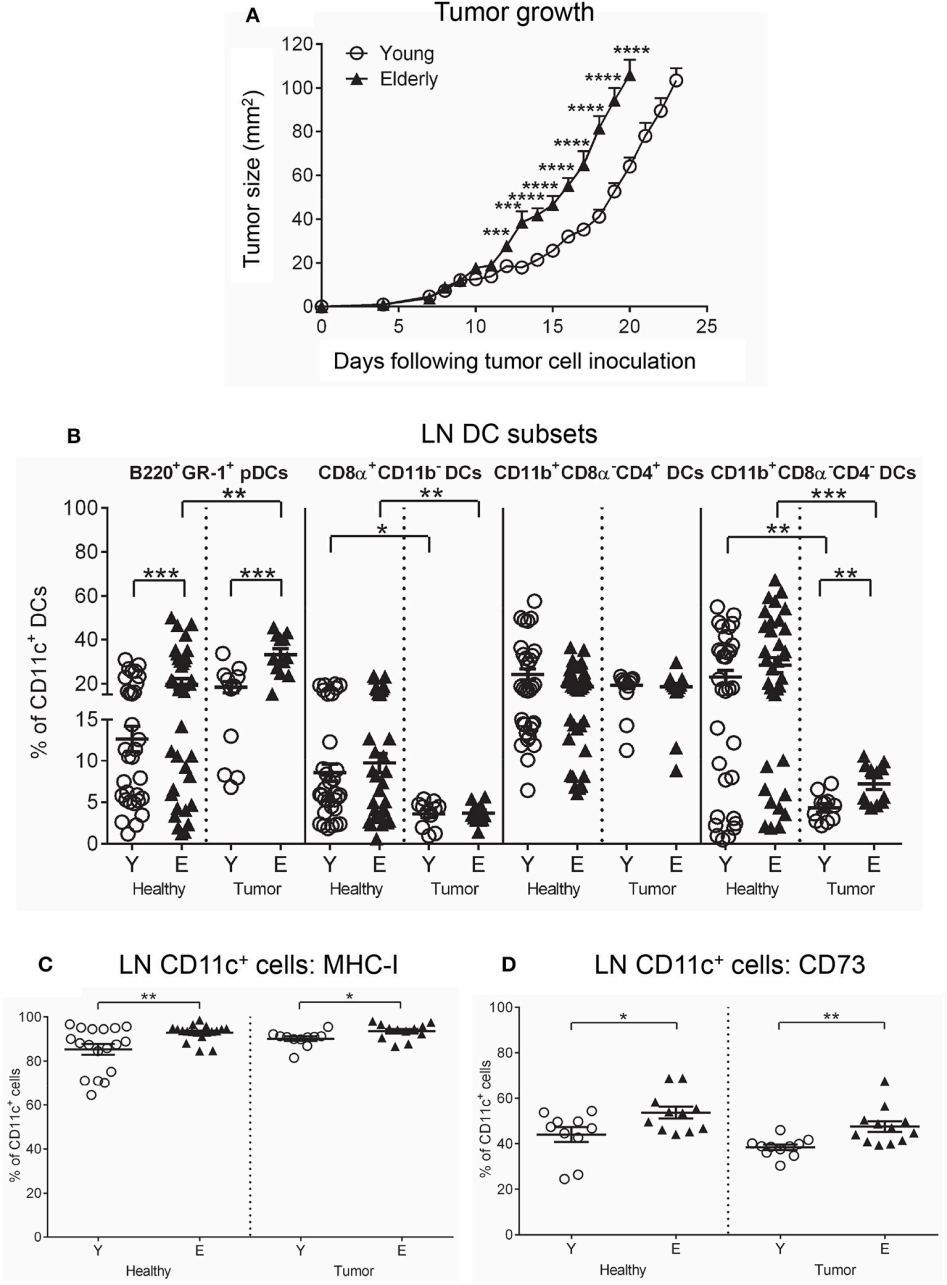
AE17 mesothelioma tumors grow faster in elderly mice. Young (*n* = 13) and elderly (*n* = 15) mice were inoculated subcutaneously with 5 × 10^5^ AE17 tumor cells per mouse, and tumor size (mm^2^) measured daily **(A)**; data shown as mean ± SEM. At end point, TDLNs from the same tumor-bearing mice and LNs from age-matched healthy mice (*n* = 32 young and 35 elderly mice) were analyzed for DC subsets **(B)**, MHC-I^+^ CD11c^+^ cells **(C)** and CD73^+^ CD11c^+^ cells **(D)** using flow cytometry. Data are shown for individual mice as well as mean ± SEM. Mouse numbers for B-D differed on account of varying cell numbers available for analysis and ranged from *n* = 11–13 young and *n* = 12–15 elderly tumor-bearing mice, *n* = 10–32 young and *n* = 11–35 elderly healthy mice, *p < 0.05, **p < 0.005, ***p < 0.0005, ****p < 0.0001 comparing (i) young to elderly mice, or (ii) tumor-bearing mice to age-matched healthy mice.

We first investigated whether aging influenced AE17 mesothelioma tumor growth in young (2–5 months) vs. elderly (20–24 months) C57BL/6J mice, and found that tumors grew faster in elderly mice (Figure 1A) (36). One possibility to account for faster tumor growth is that aging programs a more suppressive immune environment that is reflected by DCs and T cells.

### Cross-presenting DCs decrease and pDCs increase in elderly tumor-bearing lymph nodes

To determine if aging reprograms suppressive DCs, we compared overall CD11c^+^ cells (as these antigen presenting cells represent DCs) and well-described DC subsets in lymph nodes (LNs) and tumor-draining LNs (TDLNs) from young to elderly healthy and AE17 mesothelioma-bearing mice using flow cytometry (Supplementary Figures S1A–S1F).

Neither age nor the presence of a tumor significantly affected TDLN/LN CD11c^+^ cell proportions (Supplementary Figure S1G). However, examination of DC subsets showed that mesothelioma significantly decreased cross presenting CD8α^+^CD11b^−^ cDC (Figure 1B) and T helper 2 (Th2) cell-activating CD11b^+^CD8α^−^CD4^−^ cDC proportions (Figure 1B) in TDLNs compared to LNs from age-matched healthy mice. In contrast, pDC proportions already increased in healthy elderly LNs were further elevated in the presence of a tumor (Figure 1B). No age-related or tumor-induced changes were observed in CD11b^+^CD8α^−^CD4^+^ cDC proportions (Figure 1B). These data imply increased suppression due to loss of cross-presenting DCs alongside increased pDCs that are suppressive in cancer (37).

### Regulatory CD73^+^CD11C^+^ DCs increase in elderly healthy and tumor-bearing lymph nodes

To investigate whether antigen-presenting cell function is altered with aging and mesothelioma, markers of antigen presentation (MHC-I and II), activation (CD40, CD80, CD86, intracellular IFN-γ, tumor necrosis factor (TNF)-α and IL-12) and regulation (CD39, CD73, A2A receptor (A2AR), A2B receptor (A2BR), programmed cell death ligand-1 (PD-L1), galectin-9 (GAL-9), intracellular IL-10, and transforming growth factor (TGF)-β latency-associated peptide) were compared on CD11c^+^ cells from young and elderly mice using flow cytometry.

Healthy elderly LNs contained elevated proportions of MHC-I^+^CD11c^+^ cells and CD11c^+^ cells co-expressing the suppressive adenosine-producing enzyme CD73, compared to their younger counterparts (Figures 1C,D) that remained unchanged in the presence of a tumor in TDLNs (Figures 1C,D). However, mesothelioma induced age-related differences in TDLNs, including elevated proportions of IL-12^+^ and IFN-γ^+^ CD11c^+^ cells in elderly TDLNs (Supplementary Figure S1H); this is despite the observation that, regardless of age, mesothelioma reduced IL-12^+^, IFN-γ^+^, and TNF-α^+^ CD11c^+^ cell proportions in young and elderly TDLNs, compared to age-matched healthy LNs (Supplementary Figure S1H). These data imply overall loss of pro-inflammatory function with progressing mesothelioma. In contrast, mesothelioma increased MHC-II^+^ and CD80^+^CD11c^+^ cells in TDLNs, relative to age-matched healthy LNs (Supplementary Figure S2A). Expression of CD80 can be a dual-edged sword as it can ligate CD28 on T cells leading to T cell activation, or it can ligate cytotoxic T lymphocyte antigen-4 (CTLA-4) leading to T cell tolerance. Mesothelioma increased PD-L1 expression on young CD11c^+^ cells to the same levels as seen in healthy and tumor-bearing elderly DCs (Supplementary Figure S2B). No other age- or tumor-induced changes were seen on LN/TDLN CD11c^+^ cells (Supplementary Figures S2A–C).

### Elderly T cells increase expression of suppressive CD73, A2BR, CTLA-4, PD-1, ICOS, LAG-3, and IL-10

As a key function of DCs is activation or tolerization of T cell responses, we examined the effects of aging and mesothelioma on T cells in LNs/TDLNs from young and elderly mice (Supplementary Figures S3A–E). CD4^+^ T cell, CD8^+^ T cell and regulatory T cell (Treg) proportions reduced in elderly healthy LNs and TDLNs, compared to young LNs/TDLNs (Supplementary Figure S3F). Tumors reduced TDLN CD4^+^ T cell proportions in both age groups (Supplementary Figure S3F), compared to healthy LNs, yet did not affect TDLN CD8^+^ T cell and Treg proportions (Supplementary Figure S3F).

**Figure 2:**
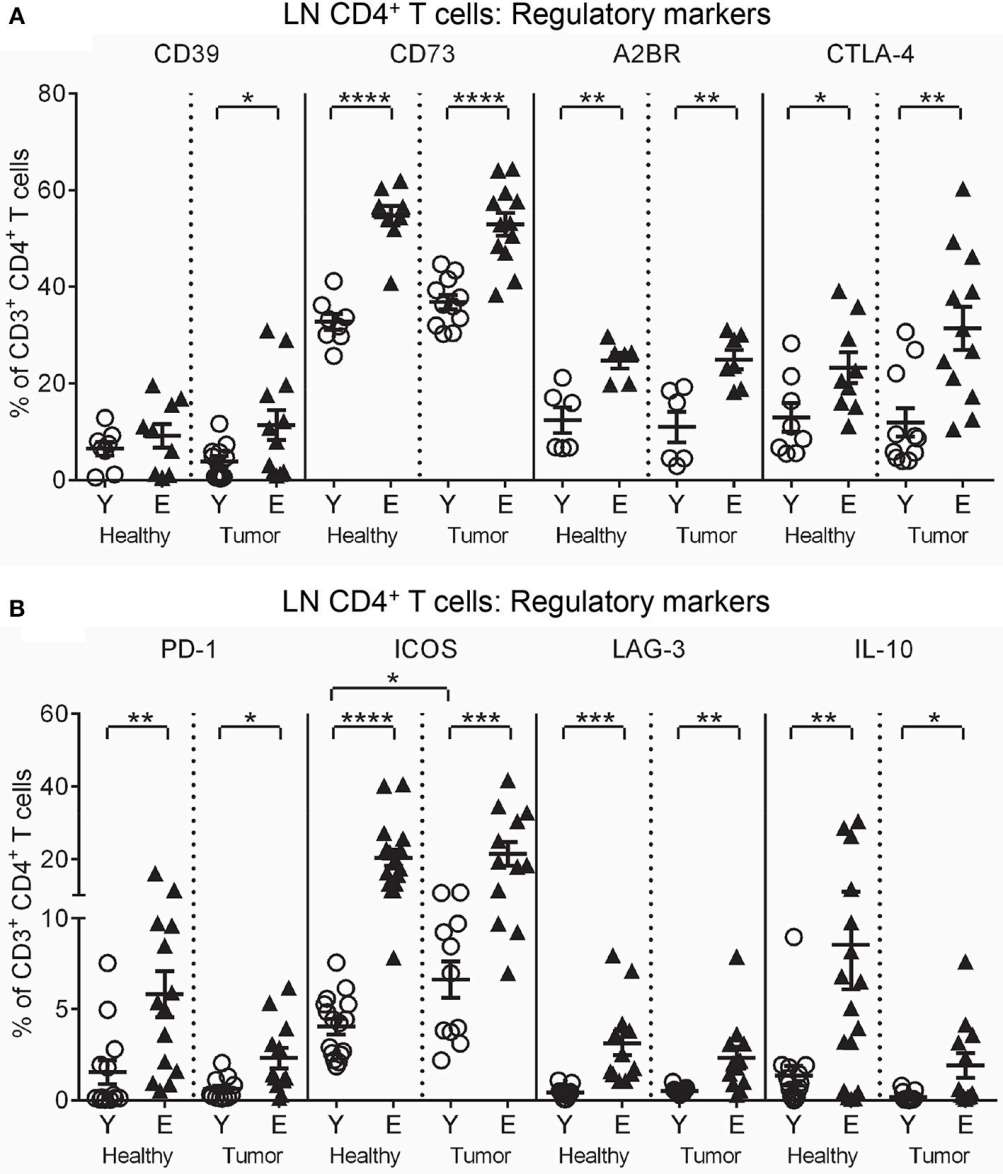
Increased regulatory markers on elderly LN/TDLN CD4^+^ T cells. Percentages of CD4^+^ T cells positive for regulatory markers were measured in TDLNs from the same mice shown in Figures 1A–D young and elderly tumor-bearing mice, and LNs from age-matched healthy young and elderly mice **(A,B)**, via flow cytometry. Data are shown for individual mice as well as as mean ± SEM, *n* = 6–11 young and *n* = 7–12 elderly tumor-bearing mice, *n* = 6–17 young and *n* = 6–17 elderly healthy mice, *p < 0.05, **p < 0.005, ***p < 0.0005, ****p < 0.0001 comparing (i) young to elderly mice, or (ii) tumor-bearing mice to age-matched healthy mice.

**Figure 3:**
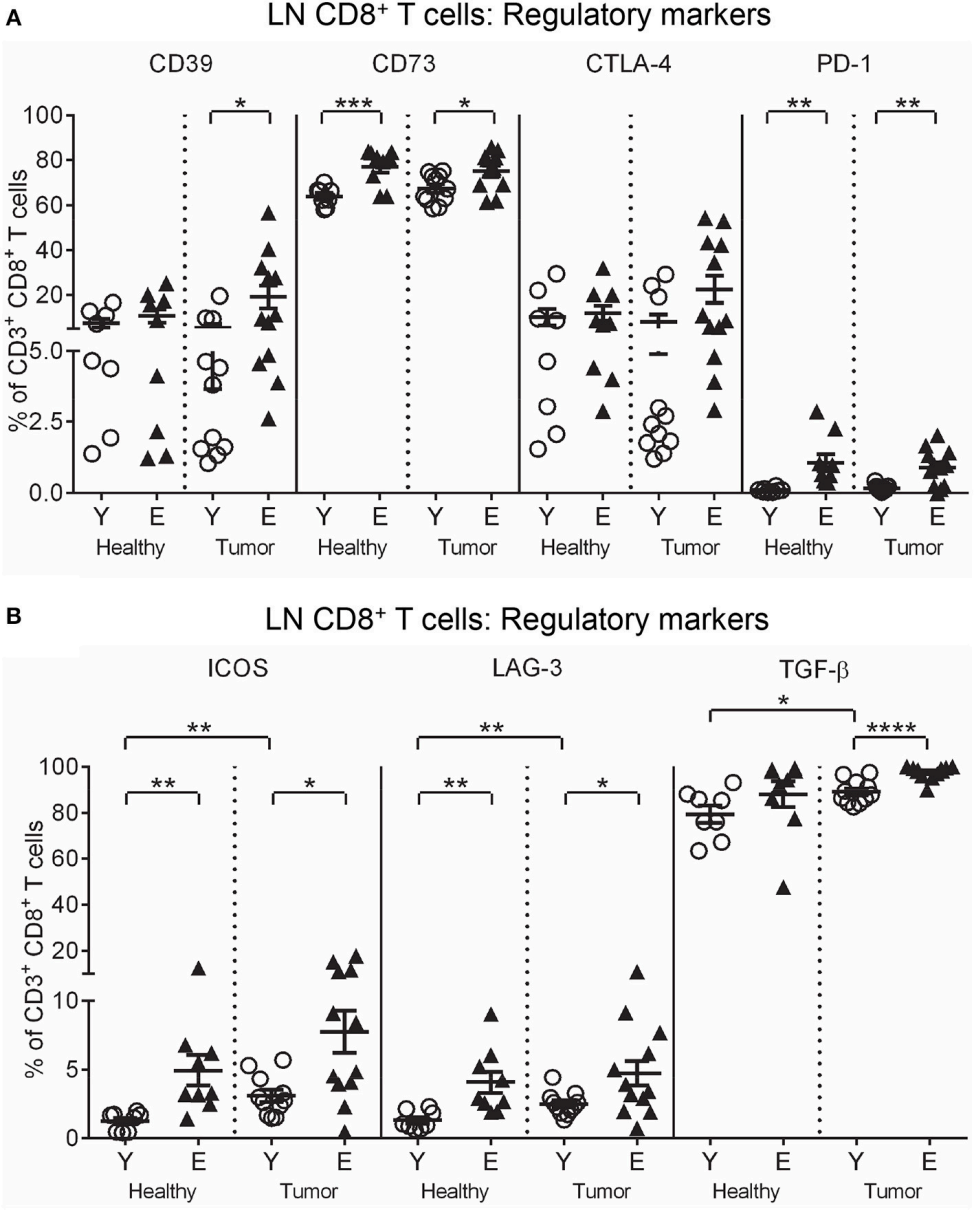
Increased regulatory markers on elderly LN/TDLN CD8^+^ T cells. Percentages of CD8^+^ T cells positive for regulatory markers were measured in TDLNs from young and elderly tumor-bearing mice, and LNs from age-matched healthy young and elderly mice, as per Figures 1, 2 (**A**, **B**), via flow cytometry. Data are shown for individual mice as well as mean ± SEM, *n* = 11 young and *n* = 12 elderly tumor-bearing mice, *n* = 8 young and *n* = 9 elderly healthy mice, *p < 0.05, **p < 0.005, ***p < 0.0005, ****p < 0.0001 comparing (i) young to elderly mice, or (ii) tumor-bearing mice to age-matched healthy mice.

To investigate function, T cells were stained for markers of activation (CD25, intracellular IFN-γ and perforin), and regulation (CTLA-4, programmed cell death protein-1 (PD-1), inducible T cell co-stimulator (ICOS), lymphocyte activation gene-3 (LAG-3), CD39, CD73, A2AR, A2BR, and intracellular IL-10 and TGF-β latency-associated peptide) and analyzed via flow cytometry. Elderly healthy LN and TDLN CD8^+^ and CD4^+^ T cells significantly increased expression of several regulatory markers, including CD39, CD73, A2BR, CTLA-4, PD-1, ICOS, LAG-3, IL-10, and TGF-β, compared to their younger counterparts (Figures 2A,B, 3A,B). Note that the presence of a tumor did not alter healthy age-related increases in regulatory markers on elderly LN/TDLN T cells (Figures 2A,B, 3A,B). This suggests that the aging environment induces LN T cells with suppressive, rather than effector, function.

The data above and summarized in Table 2 shows that LNs adopt a regulatory profile with healthy aging, characterized by increased: (i) pDCs, (ii) CD73 on CD11c^+^ DCs, and (iii) multiple regulatory markers on T cells. Furthermore, although pro-inflammatory CD11b^+^CD8α^−^CD4^−^ cDCs and IFN-γ^+^ and IL-12^+^ CD11c^+^ DC proportions were elevated in elderly TDLNs, suggesting increased T cell-activating potential, this is undermined by concomitant maintenance of age-related increases in suppressive DCs and T cells.

**Table 2:** Changes to regulatory molecules on LN CD4+ (A) and LN CD8+ (B) T cells with age and IL-2/CD40.

**(A) LN CD4**+						
	**Y healthy CD4**+	**E healthy CD4**+	**Ytumor CD4**+	**E tumor CD4**+	**Y IL-2/CD40CD4**+	**E IL-2/CD40CD8**+
CD39	6.5	9.2	3.9	11.5	7.0	15
CD73	32.7	54.7	36.9	52.9	36.1	43.7
A2BR	12.4	24.7	11.0	24.9	16.2	26.1
CTLA-4	13.0	23.3	12.0	31.4	33.2	50.7
PD-1	1.6	5.8	0.6	2.3	2.3	3.3
ICOS	4.1	20.3	6.6	21.5	21.8	48.1
LAG-3	0.4	3.1	0.5	2.3	1.9	3.9
IL-10	1.4	8.6	0.2	1.9	0.5	1.2
TGFP	82.6	88	89.9	94.4	58.5	86.8
**(B) LN CD8**+						
	**Y healthy CD8**+	**E healthy CD8**+	**Y tumor CD8**+	**E tumor CD8**+	**Y IL-2/CD40 CD8**+	**E IL-2/CD40 CD8**+
CD39	7.50	10.7	5.4	19.1	10.1	20.5
CD73	63.6	77.1	67.3	75.2	57.4	46.15
CTLA-4	10.1	11.9	8.09	22.5	23.3	34
PD-1	0.1	1.1	0.17	0.9	1.3	7.1
ICOS	1.2	4.9	3.08	7.7	15.31	30.9
LAG-3	1.3	4.1	2.48	4.7	6.3	12.8
TGFP	79.7	88.02	89.02	97.6	60.7	91.1

*The means of percentages of LN CD4+ and CD8+ T cells positive for each marker from young (Y) and elderly (E) mice, that are; healthy, tumor-bearing, or tumor-bearing and treated with IL-2/CD40. The highest percentages relative to each treatment group are highlighted by the darker shade, with a lighter shade showing the second highest levels. T cells from elderly tumor-bearing mice treated with IL-2/CD40 expressed higher levels of most regulatory markers*.

### The elderly tumor microenvironment reduces MHC-I/II and CD80 on DCs and IFN-γ in T cells.

**Figure 4:**
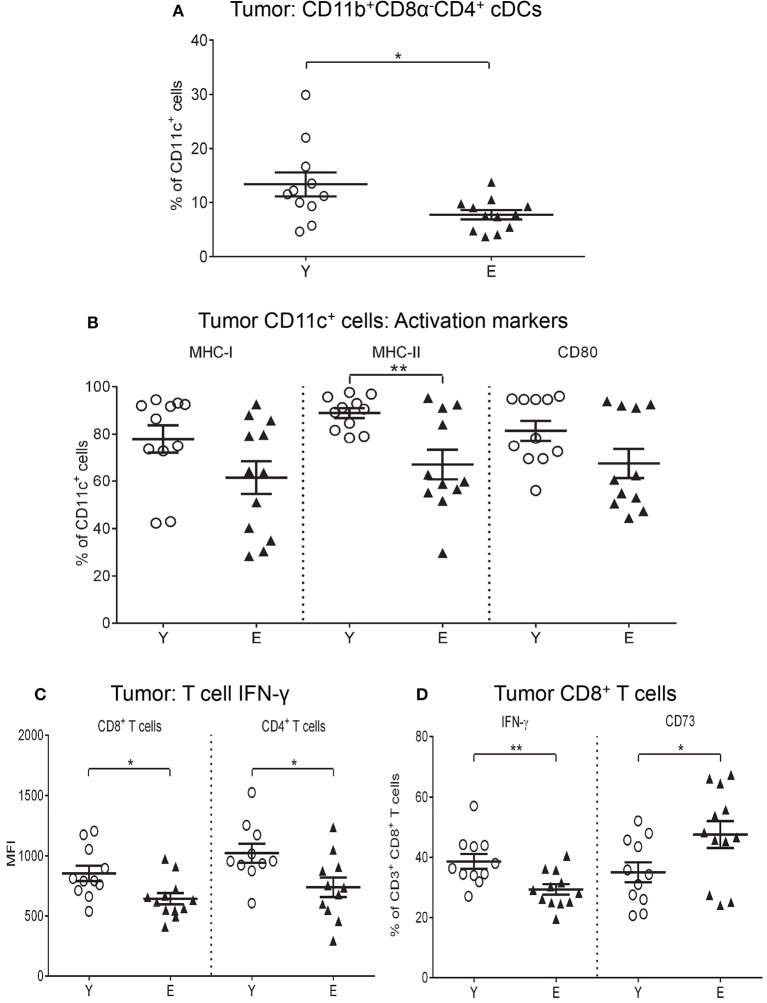
Reduced CD11b^+^CD8α^−^CD4^+^ cDCs, MHC-I/II, and CD80 on CD11c^+^ cells, and IFN-γ on T cells in elderly tumors. Young and elderly tumors as per Figures 1, 2 were analyzed using flow cytometry for percentages of CD11b^+^CD8α^−^CD4^+^ cDCs **(A)**, percentages of CD11c^+^ cells positive for MHC-I/II and CD80 **(B)**, IFN-γ expression (shown as geometric mean fluorescence intensity; MFI) on CD8^+^ and CD4^+^ T cells **(C)**, and percentages of CD8^+^ T cells positive for IFN-γ and CD73 **(D)**. Data are shown for individual mice as well as mean ± SEM, *n* = 11 young and *n* = 12 elderly tumor-bearing mice, *p < 0.05, **p < 0.005 comparing young to elderly mice.

We next investigated the tumor microenvironment and found that elderly tumors contained significantly lower levels of Th1-activating CD11b^+^CD8α^−^CD4^+^ cDCs compared to young tumors (Figure 4A). No age-related differences were seen in the other DC subsets (Supplementary Figure S4A). Elderly tumor-associated CD11c^+^ cells significantly reduced MHC-II and CD80 (Figure 4B), with a trend for reduced MHC-I (*p* = 0.08; Figure 4B) relative to their younger counterparts. In contrast, TNF-α^+^CD11c^+^ cells increased in elderly, compared to young, tumors (Supplementary Figure S4B). Examination of regulatory markers showed that IL-10^+^CD11c^+^ cells increased (Supplementary Figure S4C), whilst CD39^+^, PD-L1^+^, and TGF-β^+^ CD11c^+^ cells decreased in elderly tumors (Supplementary Figure S4C). These data suggest that aging induces deficits in elderly tumor-infiltrating CD11c^+^ cells leading to reduced antigen-presenting capacity and increased IL-10-mediated suppression.

An investigation of tumor-infiltrating T cells showed that CD4^+^ T cells and Tregs were significantly decreased in elderly mice (Supplementary Figure S5A), whilst CD8^+^ T cells were similar with age (Supplementary Figure S5A). Reduced IFN-γ was seen in CD8^+^ (Figures 4C,D) and CD4^+^ T cells (Figure 4C) in elderly tumors compared to young tumors implying reduced anti-tumor effector T cell activity with age. Examination of regulatory markers showed increased CD73^+^CD8^+^ T cells (Figure 4D), yet reduced TGF-β^+^CD8^+^ T cells (Supplementary Figure S5B), ICOS^+^CD4^+^ T cells (Supplementary Figure S5C) and TGF-β^+^CD4^+^ T cells (Supplementary Figure S5C) in elderly, compared to young tumors (data also summarized in Table 2), implying different regulatory age-related mechanisms.

### IL-2/agonist anti-CD40 immunotherapy is less effective in elderly mice

**Figure 5:**
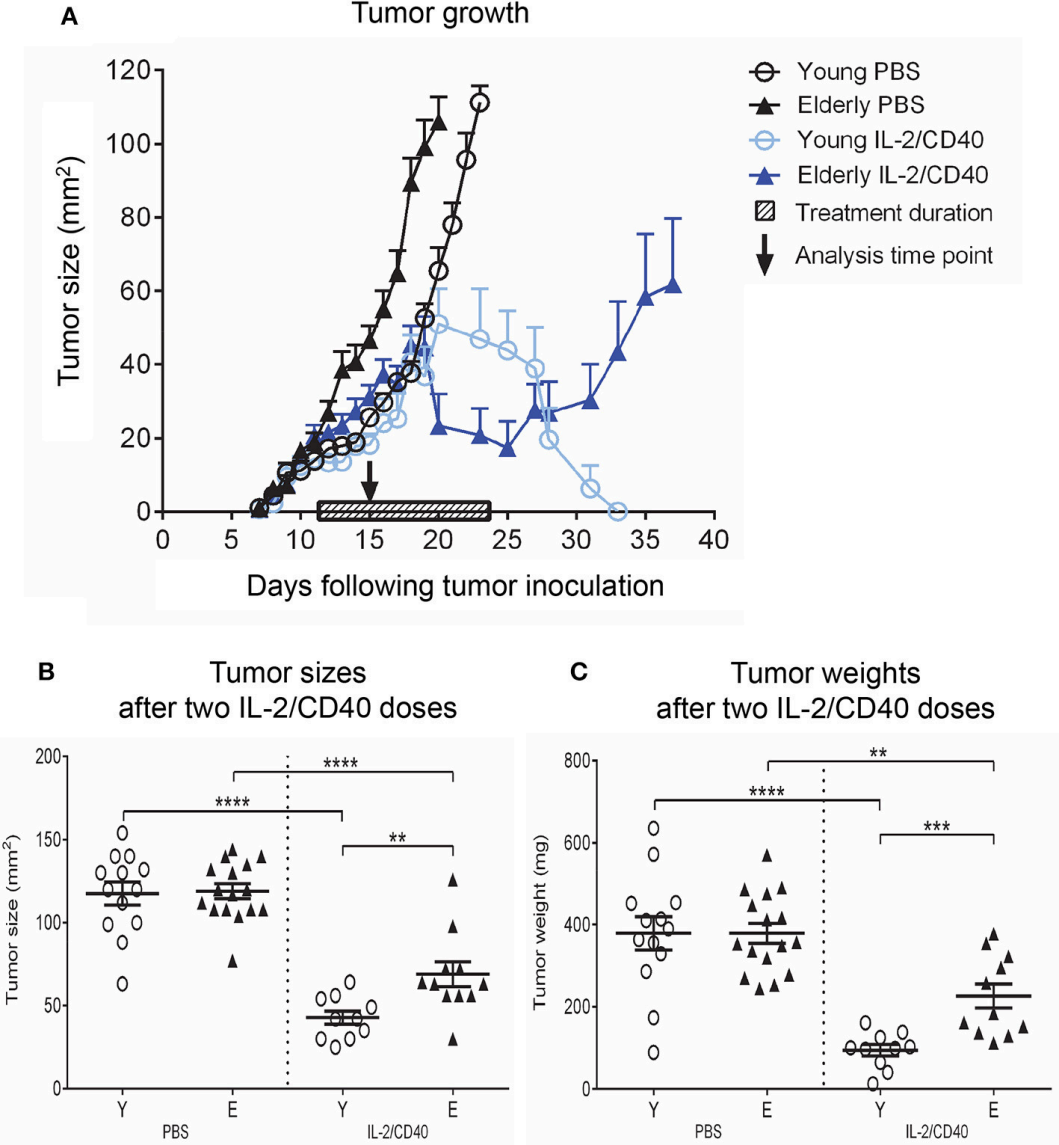
IL-2/CD40 is less efficient at inducing tumor regression in elderly mice. Young (*n* = 24) and elderly (*n* = 27) mice were inoculated subcutaneously with 5 × 10^5^ AE17 cells, and tumors left to develop before treatment with intra-tumoral IL-2 (20 μg/dose) and agonist anti-CD40 antibody, 40 μg/dose; *n* = 10 young and 11 elderly mice or PBS diluent (*n* = 14 young and 16 elderly mice; 100 μl/dose) for 5 doses, each 2–3 days apart. The shaded bar in **(A)** represents treatment duration. Tumor size (in mm^2^) was measured to determine tumor growth rates and response to IL-2/CD40 **(A)**; data shown as mean ± SEM. In a second experiment TDLNs and tumors were collected for analysis 2–3 days after the second dose, indicated by the black arrow in **(A)**, and tumor sizes **(B)** and tumor weights **(C)** in *n* = 10 young and 11 elderly mice given IL-2/CD40 and *n* = 14 young and 16 elderly mice given PBS measured; data are shown for individual mice as well as mean ± SEM, mouse numbers for C differed on account of varying cell numbers available for analysis and ranged from *n* = 10–13 young and *n* = 8–16 elderly PBS control mice, **p < 0.005, ***p < 0.0005, ****p < 0.0001 comparing (i) young to elderly mice, or (ii) IL-2/CD40-treated mice to age-matched PBS control mice.

Our previous studies demonstrated that IL-2/CD40 immunotherapy induces complete and permanent tumor regression in young mice (26, 29, 30). Herein, we investigated the efficacy of IL-2/CD40 treatment in elderly tumor-bearing mice. Young and elderly AE17 mesothelioma-bearing C57BL/6J mice were treated with IL-2/CD40, using previously described doses (26). A complete schedule of IL-2/CD40 (i.e., 5 doses) was less effective in elderly mice (45% tumor regression; Figure 5A), compared to young mice (100% tumor regression; Figure 5A) (36). Tumors and TDLNs were sampled after mice received one-third of the treatment schedule (i.e., two doses of IL-2/CD40; Figure 5A), to enable identification of tumors responding to treatment and still have enough tumor sample to collect for analysis, as young mice given the full regimen demonstrate complete resolution of tumors which therefore cannot be sampled (26, 29, 30). After two doses, IL-2/CD40 slowed tumor growth rates in both age groups, although the effects in terms of tumor size (Figure 5B) and weights (Figure 5C) were more pronounced in young mice, relative to PBS diluent controls.

### IL-2/CD40 reduces potential licensing of tumor-specific T cells by increasing CD73 and TGF-β and reducing CD40 on elderly but not young TDLN DCs

Examination of DCs in IL-2/CD40-treated mice showed that regardless of age CD11b^+^CD8α^−^CD4^+^ cDCs and CD11b^+^CD8α^−^CD4^−^ cDCs (Supplementary Figure S6A) increased relative to controls, suggesting increased capacity to stimulate Th1 and Th2 responses. No significant changes were seen in the other DC subsets (Supplementary Figure S6A).

**Figure 6:**
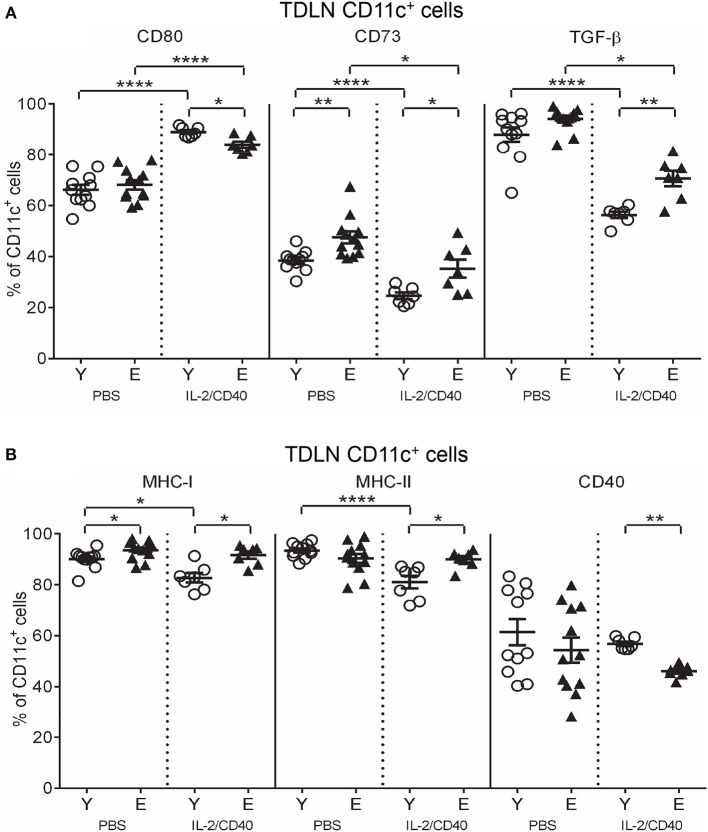
IL-2/CD40 increases MHC-I/II, CD80, CD73, and TGF-β on TDLN CD11c^+^ cells. After two doses of IL-2/CD40 or PBS diluent, percentages of CD11c^+^ cells positive for CD80, CD73, and TGF-β **(A)** and MHC-I, MHC-II,. and CD40 **(B)** were measured in TDLNs from the young and elderly tumor-bearing mice described in Figures 5B,C using flow cytometry. Data are shown as mean ± SEM, *n* = 7 young and *n* = 7 elderly IL-2/CD40-treated mice, *n* = 11 young and *n* = 12 elderly PBS control mice, *p < 0.05, **p < 0.005, ****p < 0.0001 comparing (i) young to elderly mice, or (ii) IL-2/CD40-treated mice to age-matched PBS control mice.

In both age groups, IL-2/CD40 exerted positive and negative effects on activation/maturation markers on TDLN CD11c^+^ cells: i.e., increased CD80 (Figure 6A) and CD86 (Supplementary Figure S6B), yet reduced IFN-γ, TNF-α and IL-12 (Supplementary Figure S6B), relative to age-matched PBS controls. As a result of these changes, elderly IL-2/CD40-treated mice expressed lower levels of CD80 on CD11c^+^ cells compared to their younger counterparts (Figure 6A), whilst CD86, IFN-γ, TNF-α, and IL-12 expression levels were similar for the two age groups (Supplementary Figure S6B). Importantly, IL-2/CD40 reduced expression of a number of regulatory markers on young and elderly TDLN CD11c^+^ cells including: CD73 (Figure 6A), TGF-β (Figure 6A), CD39 (Supplementary Figure S6C) and IL-10 (Supplementary Figure S6C), relative to age-matched untreated tumor-bearing mice. Nonetheless, CD73 (Figure 6A) and TGF-β (Figure 6A) remained higher on elderly CD11c^+^ cells in IL-2/CD40-treated mice. This suggests that whilst IL-2/CD40 reduces tumor-induced suppressive TDLN CD11c^+^ cells, it does not overcome the age-related increase in suppressive CD11c^+^ cells, which may be a contributing factor to the reduced efficacy of IL-2/CD40 in elderly mice.

Some age-specific effects were also seen. Young, but not elderly, IL-2/CD40-treated mice down-regulated MHC-I (Figure 6B) and MHC-II (Figure 6B) on DCs, relative to age-matched PBS controls resulting in an increased age-related differential (Figure 6B). In contrast, CD40 on TDLN CD11c^+^ cells reduced (Figure 6B) in elderly, compared to young, IL-2/CD40-treated mice. Taken together, whilst CD11c^+^ cells from elderly IL-2/CD40-treated mice have increased capacity to present antigens to T cells, their ability to license tumor-specific T cells appears compromised, relative to young counterparts.

### IL-2/CD40 further exacerbates the regulatory status of elderly TDLN T cells

The effects of IL-2/CD40 on CD8^+^ and CD4^+^ T cells and Tregs in TDLNs and tumors were also examined. IL-2/CD40 reduced TDLN CD4^+^ T cell proportions in young and elderly mice, relative to age-matched PBS controls (Supplementary Figure S7A). Unexpectedly, young IL-2/CD40-treated mice showed a reduction in TDLN CD8^+^ T cell proportions relative to their PBS controls (Supplementary Figure S7A).

**Figure 7:**
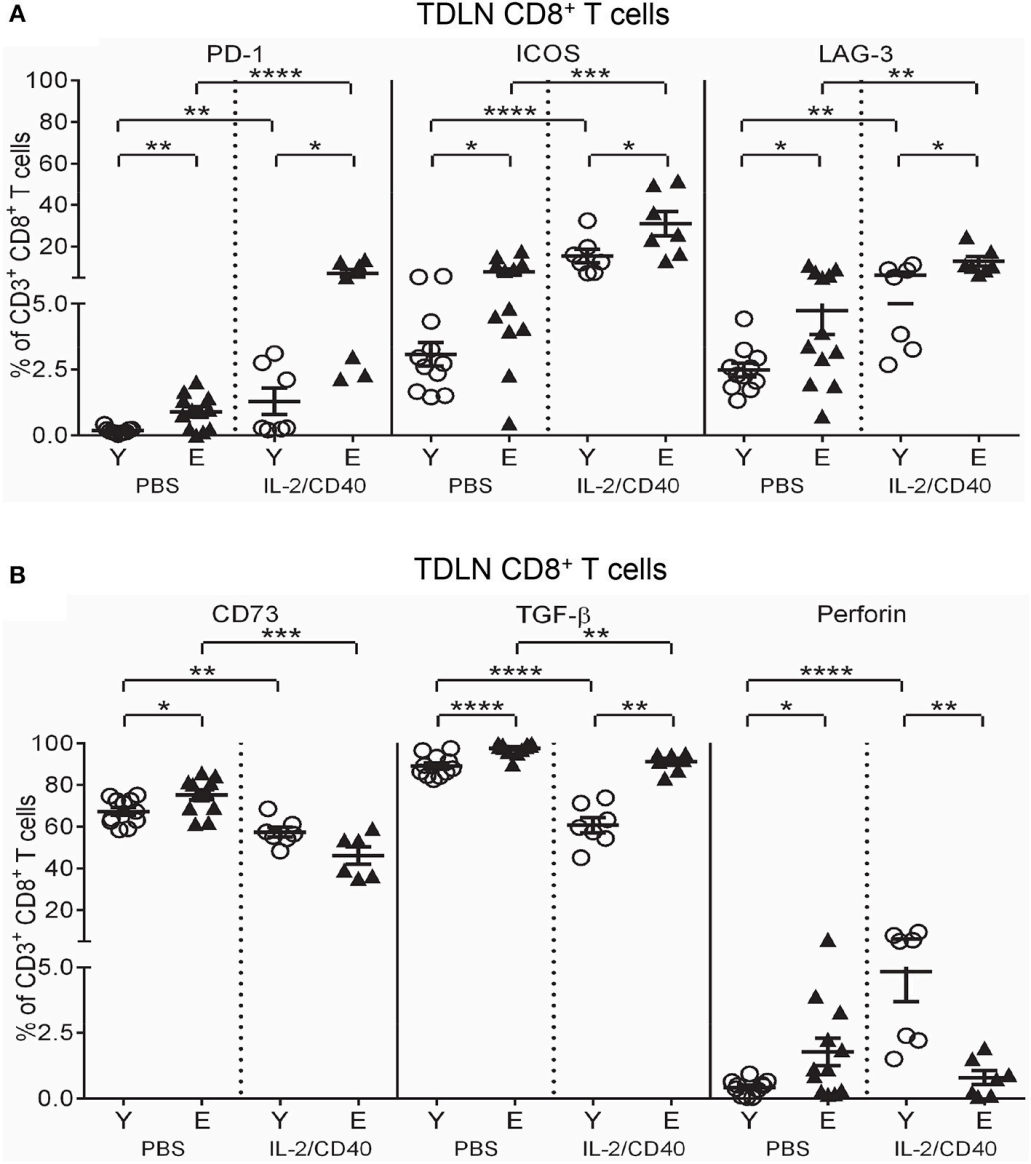
IL-2/CD40 increases PD-1, ICOS, LAG-3, CD73, and TGF-β on TDLN CD8^+^ T cells. After two doses of IL-2/CD40 or PBS diluent, percentages of CD8^+^ T cells positive for PD-1, ICOS, and LAG-3 **(A)** and CD73, TGF-β and perforin **(B)** were measured in TDLNs from the young and elderly tumor-bearing mice described in Figures 5B and C using flow cytometry. Data are shown as mean ± SEM, *n* = 7 young and *n* = 6–7 elderly IL-2/CD40-treated mice, *n* = 11 young and *n* = 12 elderly PBS control mice, *p < 0.05, **p < 0.005, ***p < 0.0005, ****p < 0.0001 comparing (i) young to elderly mice, or (ii) IL-2/CD40-treated mice to age-matched PBS control mice.

IL-2/CD40 exerted positive and negative effects on regulatory marker expression on TDLN CD8^+^ T cells in both age groups, including increased PD-1, ICOS, and LAG-3 (Figure 7A), compared to age-matched PBS controls. Nonetheless, age-related differences in PD-1, ICOS, and LAG-3 were maintained, whereby elderly CD8^+^ T cells had higher expression (Figure 7A). In contrast, IL-2/CD40 induced reduced CD73 and TGF-β expression on CD8^+^ T cells (Figure 7B) in young and elderly TDLNs, relative to age-matched PBS controls. However, IL-2/CD40-treated young mice demonstrated a striking reduction in TGF-β, meaning elderly mice still expressed significantly higher TGF-β (Figure 7B). Overall, elderly TDLN CD8^+^ T cells maintain increased suppressive function with IL-2/CD40 treatment.

Interestingly, IL-2/CD40 exerted age-specific effects on TDLN CD8^+^ T cell perforin expression, with young mice up-regulating perforin expression (Figure 7B), resulting in higher expression compared to elderly IL-2/CD40-treated mice (Figure 7B). This suggests that in addition to their increased regulatory status, elderly TDLN CD8^+^ T cells have reduced cytotoxic effector activity due to a failure to up-regulate perforin in response to IL-2/CD40.

**Figure 8:**
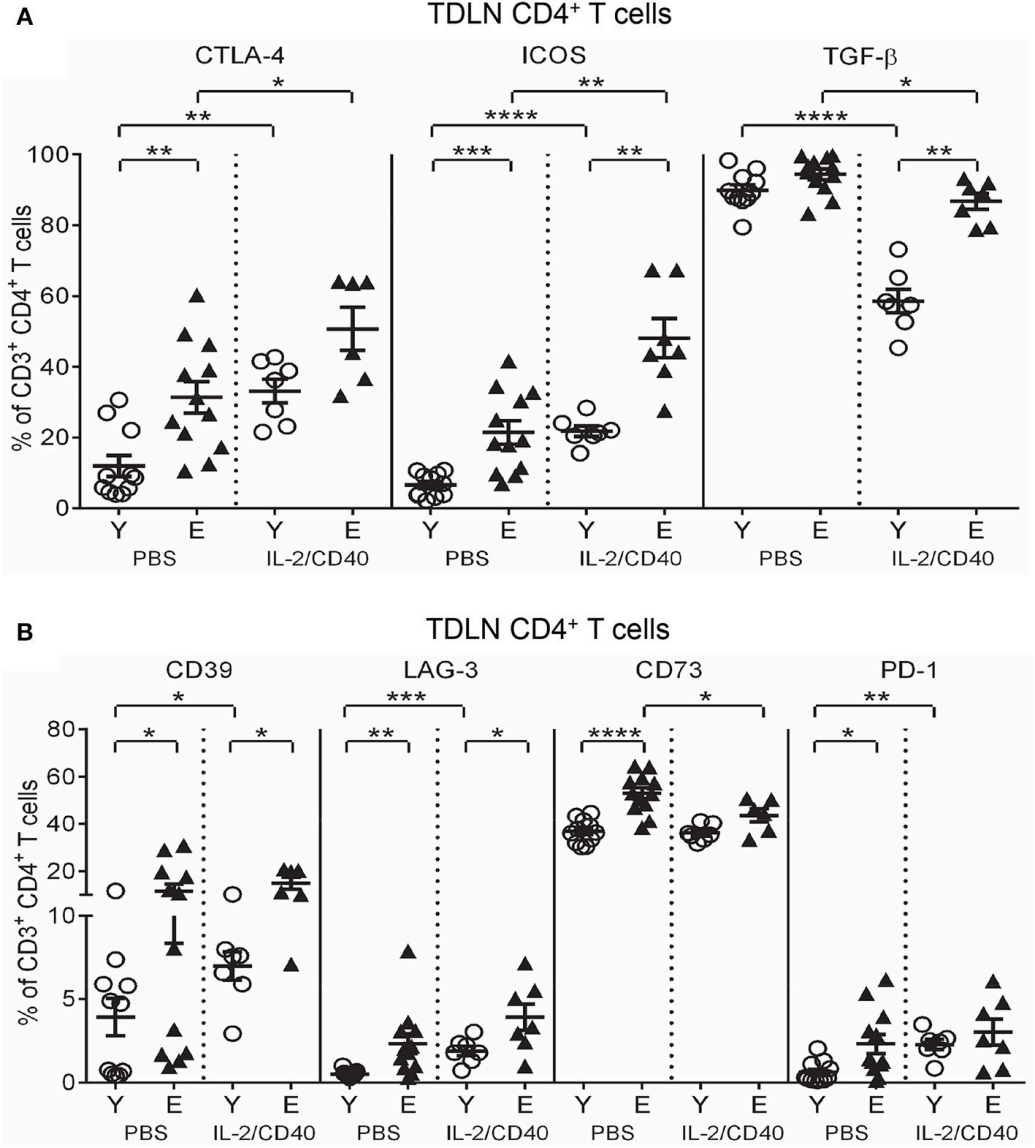
IL-2/CD40 increases CTLA-4, ICOS, TGF-β, CD39, and LAG-3 on young and elderly TDLN CD4^+^ T cells. After two doses of IL-2/CD40 or PBS diluent, percentages of CD4^+^ T cells positive for CTLA-4, ICOS and TGF-β **(A)** and CD39, LAG-3, CD73, and PD-1 **(B)** were measured in TDLNs from the young and elderly tumor-bearing mice described in Figures 5B,C using flow cytometry. Data are shown as mean ± SEM, *n* = 7 young and *n* = 6–7 elderly IL-2/CD40-treated mice, *n* = 11 young and *n* = 12 elderly PBS control mice, *p < 0.05, **p < 0.005, ***p < 0.0005, ****p < 0.0001 comparing (i) young to elderly mice, or (ii) IL-2/CD40-treated mice to age-matched PBS control mice.

IL-2/CD40 also exerted common effects on several regulatory markers on TDLN CD4^+^ T cells from both age groups including increased CTLA-4 and ICOS and decreased TGF-β expression (Figure 8A), compared to age-matched PBS controls. Despite these changes, expression of CTLA-4, ICOS, and TGF-β remained higher on elderly TDLN CD4^+^ T cells compared to young IL-2/CD40-treated mice (Figure 8A).

Age-specific effects of IL-2/CD40 on TDLN CD4^+^ T cells were also seen. Elderly, but not young, IL-2/CD40-treated mice reduced CD4^+^ T cells expressing CD73 (Figure 8B) relative to controls. CD4^+^ T cells from young, but not elderly, IL-2/CD40-treated mice up-regulated CD39, LAG-3, and PD-1 (Figure 8B), compared to their PBS controls however, this did not alter the age differentials seen in CD39 and LAG-3 in untreated mice, with expression of these markers remaining higher on elderly CD4^+^ T cells (Figure 8B); data summarized in Tables 2. Taken together, elderly TDLN CD4^+^ T cells also demonstrate an enhanced regulatory status during IL-2/CD40 treatment, which may contribute to the reduced efficacy of IL-2/CD40 in elderly mice.

### IL-2/CD40 increases suppressive TGF-β and A2BR and reduces CD40 on DCs in elderly tumors

**Figure 9:**
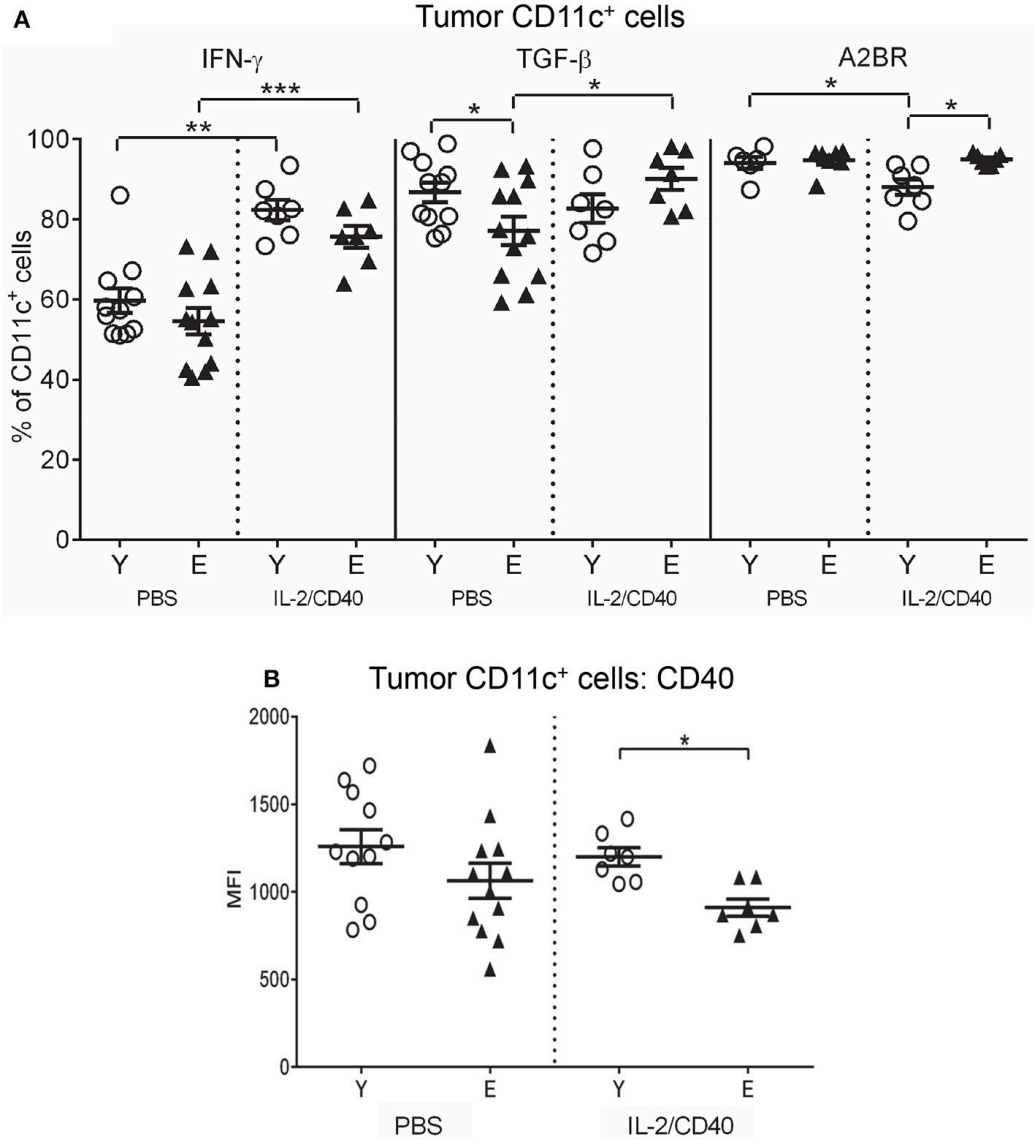
Increased IFN-γ, TGF-β, and A2BR, and reduced CD40 on CD11c^+^ cells from elderly IL-2/CD40-treated tumors. After two doses of IL-2/CD40 or PBS diluent, percentages of CD11c^+^ cells positive for IFN-γ, TGF-β and A2BR **(A)** and expression levels of CD40 (shown as geometric mean fluorescence intensity; MFI) on CD11c^+^ cells **(B)** were measured from the young and elderly tumor-bearing mice described in Figures 5B,C using flow cytometry. Data are shown as mean ± SEM, *n* = 7 young and *n* = 7 elderly IL-2/CD40-treated mice, *n* = 6–11 young and *n* = 7–12 elderly PBS control mice, *p < 0.05, **p < 0.005, ***p < 0.0005 comparing (i) young to elderly mice, or (ii) IL-2/CD40-treated mice to age-matched PBS control mice.

IL-2/CD40 mostly did not alter young or elderly tumor-associated DC subset proportions, relative to untreated mice, with the exception of a small, but significant, increase in cross-presenting CD8α^+^CD11b^−^ cDCs in elderly tumors (Supplementary Figure S7B). The only common effect of IL-2/CD40 on tumor-associated CD11c^+^ cells seen in both age groups was up-regulation of IFN-γ (Figure 9A).

IL-2/CD40 induced several age-specific changes on tumor-associated CD11c^+^ cells including increased TGF-β on elderly DCs (Figure 9A). In contrast, IL-2/CD40 induced reduced MHC-I and CD80 (Supplementary Figure S7C) in young, but not elderly, tumor-associated CD11c^+^ cells. Concurrently, IL-2/CD40 reduced expression of several regulatory markers on young CD11c^+^ cells including: CD39 (Supplementary Figure S7C), A2AR (Supplementary Figure S7D), A2BR (Figure 9A) and PD-L1 (Supplementary Figure S7D), yet expression of CD73 increased (Supplementary Figure S7D), relative to PBS controls. However, elderly CD11c^+^ cells demonstrated reduced CD40 (Figure 9B) and increased A2BR (Figure 9A) relative to young CD11c^+^ cells. Taken together, this suggests that IL-2/CD40 alleviates suppressive CD11c^+^ cells in young, but not elderly tumors, likely contributing to reduced tumor responses to IL-2/CD40 by elderly mice.

### IL-2/CD40 reduces IFN-γ and perforin on elderly tumor-infiltrating CD8^+^ T cells

Examination of tumor-associated T cells showed that IL-2/CD40 reduced young, but not elderly, CD4^+^ T cell (Supplementary Figure S8A) and Treg proportions (Supplementary Figure S8A), leaving elevated Treg proportions in elderly IL-2/CD40-treated tumors (Supplementary Figure S8A), implying increased suppressive activity in elderly tumors.

**Figure 10:**
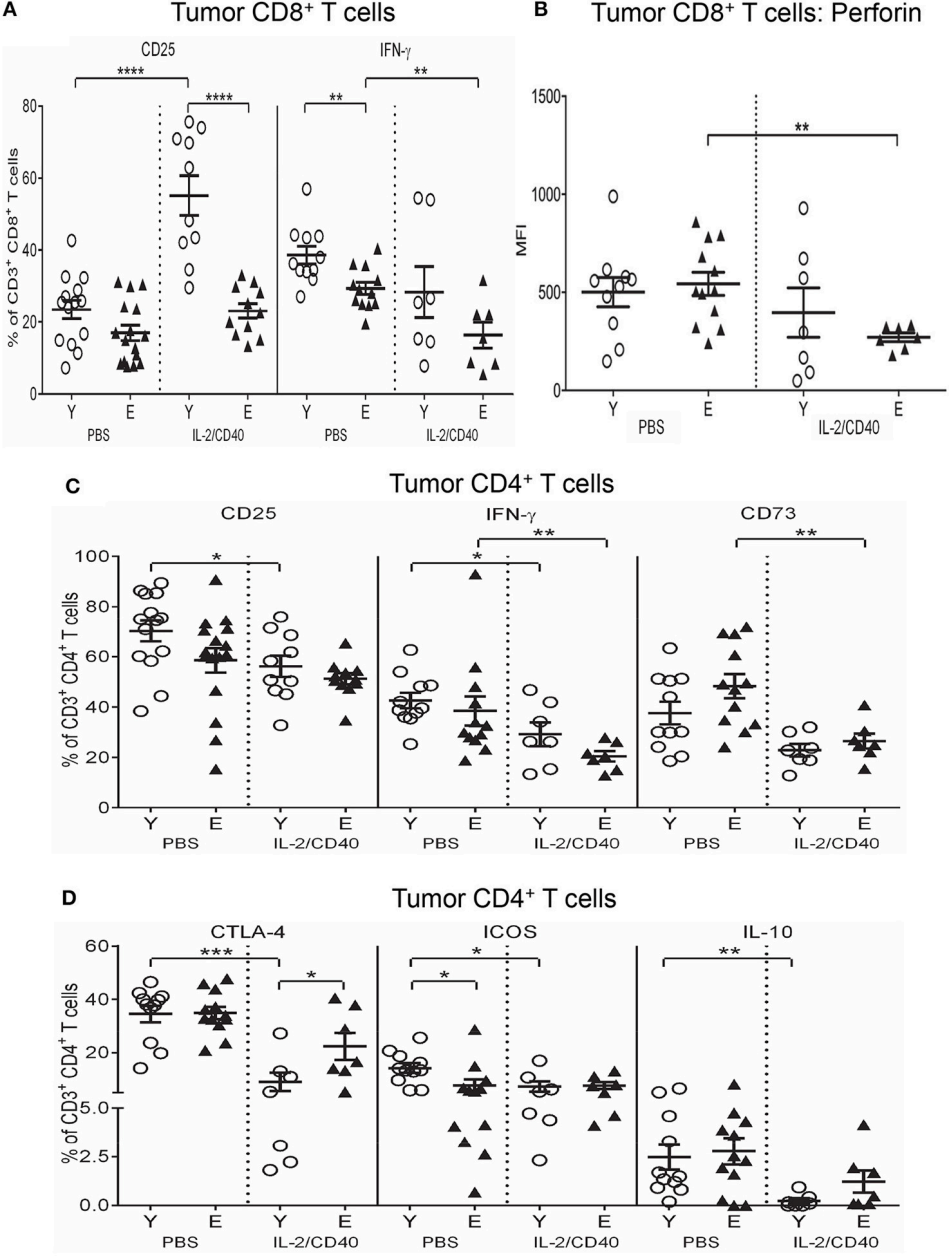
Reduced CD25, IFN-γ and perforin on CD8^+^ T cells and increased CTLA-4 on CD4^+^ T cells in elderly IL-2/CD40-treated tumors. After two doses of IL-2/CD40 or PBS diluent, percentages of CD8^+^ T cells positive for CD25 and IFN-γ **(A)**, expression levels of perforin (shown as geometric mean fluorescence intensity; MFI) on CD8^+^ T cells **(B)**, and percentages of CD4^+^ T cells positive for CD25, IFN-γ and CD73 **(C)** and CTLA-4, ICOS and IL-10 **(D)** were measured in tumors from the young and elderly tumor-bearing mice described in Figures 5B,C using flow cytometry. Data are shown as mean ± SEM, *n* = 7–10 young and *n* = 7–11 elderly IL-2/CD40-treated mice, *n* = 11–14 young and *n* = 12–16 elderly PBS control mice, *p < 0.05, **p < 0.005, ***p < 0.0005, ****p < 0.0001 comparing (i) young to elderly mice, or (ii) IL-2/CD40-treated mice to age-matched PBS control mice.

Regardless of age, IL-2/CD40 up-regulated CD25 on tumor-associated CD8^+^ T cells (Figure 10A) relative to PBS controls, this increase was more striking in young mice (Figure 10A).

Several age-specific effects of IL-2/CD40 were also observed. In elderly IL-2/CD40-treated tumors, IFN-γ^+^CD8^+^ T cells (Figure 10A) and perforin levels on CD8^+^ T cells (Figure 10B) reduced compared to controls. This was accompanied by reductions in tumor-associated CD8^+^ T cells expressing CD73 (Supplementary Figure S8B) and IL-10 (Supplementary Figure S8B) in elderly, but not young, IL-2/CD40-treated mice, relative to PBS controls. Overall, despite the observation that IL-2/CD40 alleviates certain suppressive functions in elderly tumor-infiltrating CD8^+^ T cells, these cells also demonstrated reduced anti-tumor effector function.

Unexpectedly, IL-2/CD40 promoted a more regulatory phenotype in young tumor-associated CD8^+^ T cells, on account of increases in several regulatory markers, relative to PBS controls, specifically: CD39, A2AR, A2BR, ICOS, LAG-3, and PD-1 (Supplementary Figures S8B,C). As a result, expression of CD39, A2AR, A2BR, ICOS and LAG-3 (Supplementary Figures S8B,C) was higher on tumor-associated CD8^+^ T cells from young, compared to elderly, IL-2/CD40-treated mice. These data may reflect transition of young CD8^+^ T cells to the attenuation phase of the immune response following IL-2/CD40 stimulation.

### IL-2/CD40 reduces CTLA-4, ICOS and IL-10-mediated suppressive potential in young, but not elderly, tumor-infiltrating CD4^+^ T cells

IL-2/CD40 reduced CD25 (Figure 10C) and IFN-γ (Figure 10C) in tumor-associated CD4^+^ T cells in both age groups alongside reductions in the regulatory markers CD73 (Figure 10C) and CTLA-4 (Figure 10D). The reduction in CTLA-4 was more striking for young mice (Figure 10D). Furthermore, young, but not elderly, IL-2/CD40-treated mice demonstrated additional reductions in other regulatory markers expressed by tumor-associated CD4^+^ T cells, including ICOS and IL-10 (Figure 10D). Reduction of several layers of suppression in young tumor-associated CD4^+^ T cells may help account for the improved tumor response seen in young mice to IL-2/CD40 treatment.

### Reduced tumor-specific CTL activity in elderly TDLNs and tumors is not restored by IL-2/CD40

**Figure 11:**
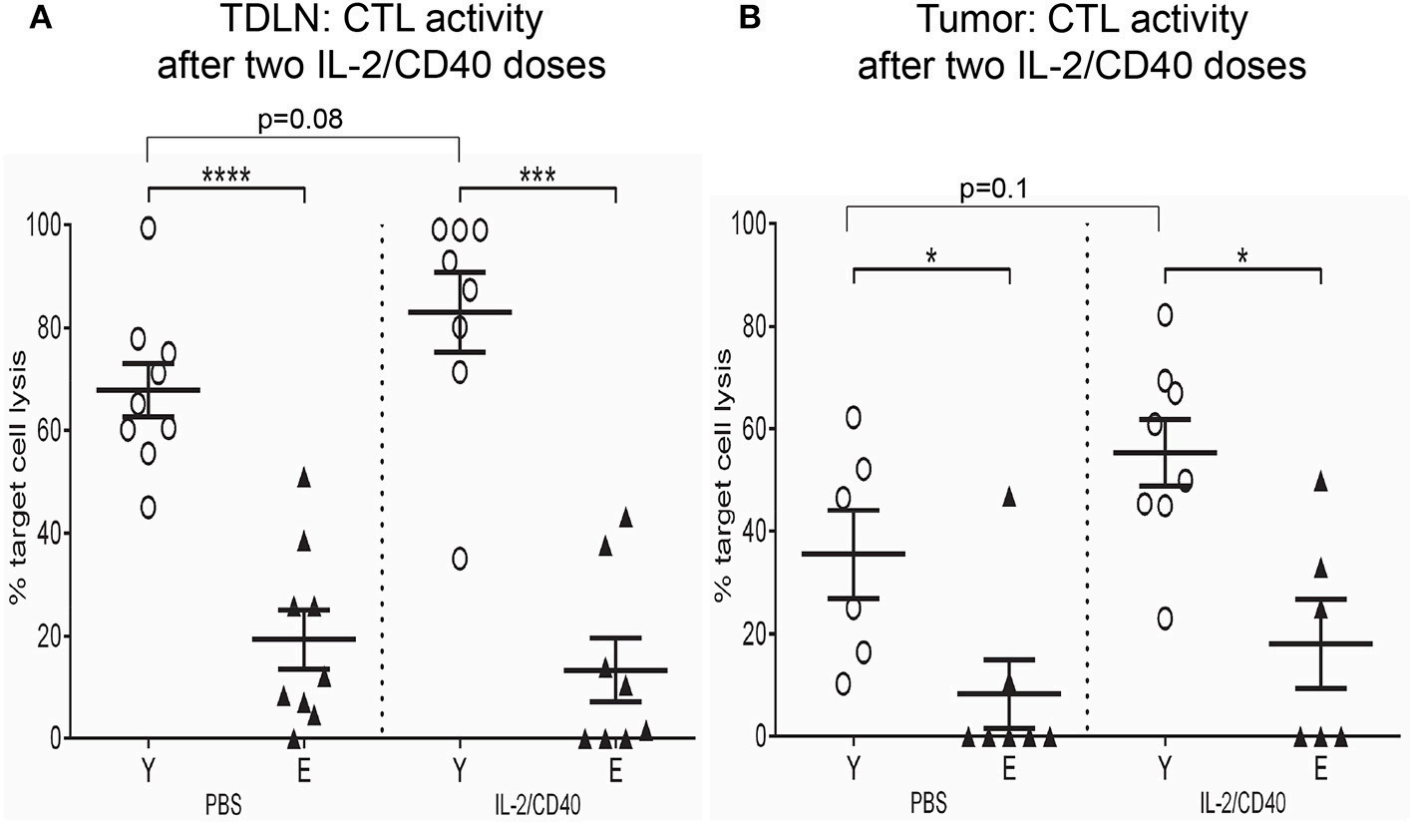
Reduced tumor-specific CTL activity in elderly TDLNs and tumors. In a further experiment the cytotoxic activity of tumor-specific CD8^+^ CTLs was assessed after two doses of IL-2/CD40 or PBS diluent in TDLNs **(A)** and tumors **(B)** of young (*n* = 17) and elderly (*n* = 17) AE17sOVA-bearing mice using an *in vivo* CTL assay. SIINFEKL-pulsed and unpulsed target cells were injected into tumor-bearing mice, and ratios between percentages of the two target cell populations in TDLNs and tumors measured 18 h later via flow cytometry. Data are shown for individual mice as well as mean ± SEM, mouse numbers differed on account of varying cell numbers available for analysis and ranged from *n* = 8 young and *n* = 6–8 elderly IL-2/CD40-treated mice, *n* = 6–9 young and *n* = 7–9 elderly PBS control mice, *p < 0.05, ***p < 0.0005, ****p < 0.0001 comparing young to elderly mice.

The data above suggests elderly CD8^+^ T cells have reduced effector function and increased suppressive function, therefore we assessed the cytolytic capacity of young vs. elderly tumor-specific CD8^+^ T cells in TDLNs and tumors after two doses of IL-2/CD40 using an *in vivo* CTL assay. This assay uses AE17sOVA-bearing young and elderly mice, where ovalbumin acts as a marker tumor antigen, enabling measurement of antigen-specific CTL responses against SIINFEKL, the dominant peptide of ovalbumin. SIINFEKL-pulsed and unpulsed control target cells were injected into young and elderly AE17sOVA-bearing mice treated with PBS diluent or IL-2/CD40. 24 h later LNs and tumors were removed and lysis of SIINFEKL-labeled target cells measured to provide an indication of tumor-specific CTL activity. Significantly reduced SIINFEKL-specific lytic activity was observed in TDLNs (Figure 11A) and tumors (Figure 11B) of elderly IL-2/CD40-treated and PBS control mice, relative to their younger counterparts (36). IL-2/CD40 induced increased trends for CTL activity in young TDLNs (*p* = 0.08; Figure 11A) and tumors (*p* = 0.1; Figure 11B) whilst no increase in CTL activity was observed in elderly IL-2/CD40-treated mice, relative to PBS controls (Figures 11A,B). The age-related reduction in tumor-specific CTL function in untreated (PBS) elderly tumor-bearing mice may account for the faster tumor growth rate seen in elderly mice. Poorer CTL responses in elderly mice is likely to contribute to their reduced tumor responses to IL-2/CD40-treatment.

## Discussion

Our study shows that aging programs development of a suppressive immune environment, which is reflected by DCs and T cells, and this may compromise generation of anti-tumor immune responses, influence mesothelioma progression and responses to IL-2/CD40 immunotherapy. We observed establishment of a suppressive environment within healthy elderly LNs, evidenced by increased regulatory pathways on DCs and T cells. This regulatory LN environment was maintained in TDLNs of elderly mesothelioma-bearing mice. As TDLNs are the main site of DC/T cell interactions leading to generation of anti-tumor effector T cell responses, increased suppressive mechanisms in elderly TDLNs likely led to compromised generation of anti-mesothelioma effector T cell responses, thereby contributing to the faster tumor growth rate observed in the elderly.

Elevated pDCs in elderly LNs/TDLNs represents one suppressive pathway that is likely to negatively affect elderly anti-tumor immune responses, as pDCs have been associated with immune suppression in cancer (37). There are several mechanisms by which pDCs induce immune suppression, including: (i) inducing Th1 cell anergy and apoptosis via indoleamine 2,3-dioxygenase (IDO) (38), (ii) inhibiting Th1 cell proliferation via granzyme B (39), and (iii) promoting suppressive Treg expansion via IDO (40, 41) and/or expression of ICOS ligand (ICOS-L), which binds ICOS expressed on Tregs, leading to Treg activation and IL-10 secretion (42–45). In turn, Tregs mediate suppression within LNs, by inhibiting effector CD8^+^ and CD4^+^ T cells, and promoting regulatory function in DCs (46). Thus, the age-related increase in LN/TDLN pDCs likely compromises the development of effective anti-mesothelioma immune responses in the elderly.

Age-related increases in several regulatory markers on LN/TDLN CD11c^+^ cells and T cells represents a further mechanism that contributes to a suppressive environment in the elderly. As the outcome of a T cell response depends on the summation of positive and negative signals it receives (47), age-related increases in regulatory molecules will skew the balance in favor of negative signals, leading to elderly T cell suppression, rather than activation. LN/TDLN CD11c^+^ cells demonstrated simultaneous increases in antigen-presenting (MHC-I), co-stimulatory (CD80, IL-12, and IFN-γ) and regulatory (CD73 and PD-L1) molecules with aging. Therefore, whilst antigen presentation to T cells may be maintained with aging, it is likely to be accompanied by an increased breadth of negative signals. The strength of a signal delivered by a molecule is determined by interaction with its ligand, and although we observed increased CD80 expression on elderly LN CD11c^+^ cells, others have reported that expression of its ligand, CD28, is reduced on elderly T cells (48–51), suggesting reduced T cell activation in the elderly. In this scenario, CD80 may instead play a role in inhibiting T cell responses during aging. This study, and others (52, 53), showed increased CTLA-4 expression on elderly T cells; ligation of CD80 and CTLA-4 negatively regulates T cell activation (54). Furthermore, CD80 on DCs can also bind PD-L1 on T cells, leading to T cell inhibition (55, 56), and others have shown that PD-L1 expression is increased on aged CD8^+^ T cells (57, 58). Thus, elevated CD80 on elderly CD11c^+^ cells may lead to T cell suppression.

The PD-L1/PD-1 pathway represents another mechanism by which T cell responses may be suppressed during aging. In agreement with others, this study observed increased PD-L1 expression on elderly LN CD11c^+^ cells (57), and increased PD-1 expression on elderly LN/TDLN T cells (52, 53, 57, 59–63), suggesting that the likelihood of inhibitory PD-L1/PD-1 interactions is increased with aging. The outcomes of this include inhibition of T cell proliferation, reduced effector T cell differentiation, reduced cytokine production and cytotoxic function, as well as increased T cell apoptosis (64–67), thereby compromising elderly effector T cell responses.

In addition, the age-related increase in LAG-3 expression on T cells, also observed in another study (62), may lead to increased negative signaling through the MHC-II/T cell receptor complex (68), and could contribute to the impaired ability of elderly DCs to prime CD4^+^ T cells and Th1 responses, described by others (7, 69, 70).

Increased CD73 on elderly CD11c^+^ cells represents another mechanism of T cell suppression as it indicates increased capacity to produce immunosuppressive adenosine, particularly if neighboring cells express CD39 (71), such as the concomitant increase in CD39 expression we observed on elderly TDLN T cells. Elderly T cells also had increased CD73, suggesting they contribute to the generation of adenosine. Moreover, elderly CD4^+^ T cells in LNs/TDLNs have an increased capacity to respond to adenosine due to elevated expression of the adenosine-binding A2B receptor. Adenosine-mediated signaling deactivates T cells by impairing IL-2 production (72, 73) and inducing Tregs (74, 75). Taken together, our data suggests that with aging, negative signals outweigh positive signals during DC/T cell interactions in elderly LN/TDLNs, leading to suppression of tumor-specific effector T cells. This is supported by our observation that CTLs in elderly TDLNs displayed reduced tumor antigen-specific cytolytic activity relative to their younger counterparts. Taken together, these changes suggest diminished anti-mesothelioma immune responses in the elderly, contributing to faster tumor progression.

The age-induced up-regulation of several inhibitory markers on LN/TDLN T cells not only suggests an increased capacity to respond to negative signals from DCs, but also implies that elderly T cells are functionally exhausted. In agreement with others, we observed that elderly CD8^+^ and CD4^+^ T cells had increased PD-1, CTLA-4, ICOS, LAG-3, and CD39 expression (52, 53, 57, 59–63, 76, 77), with simultaneous up-regulation of checkpoint inhibitory markers (particularly PD-1, CTLA-4, LAG-3, TIM-3, and CD39) being a hallmark of exhausted T cells (78, 79). This regulatory phenotype was accompanied by reduced lytic effector function in elderly CD8^+^ T cells. T cell exhaustion has been described in cancer (80–82) and is a state in which T cells are unable to proliferate, produce lower levels of cytokines such as IL-2 and IFN-γ, have diminished cytotoxic activity and effector function, and cannot generate memory responses (78, 83). The development of an exhausted phenotype in aged T cells is supported by studies showing that PD-1-expressing elderly T cells are dysfunctional, as they have reduced proliferative capacity (53, 57, 59, 63). Thus, aging-induced development of functionally exhausted T cells with diminished anti-tumor effector activity represents another potential mechanism contributing to faster mesothelioma tumor growth in the elderly.

Examination of CD11c^+^ cells and T cells within tumors provided further evidence that a suppressive immune environment develops during aging. CD11c^+^ cells in elderly tumors may have reduced ability to present antigens and stimulate CD8^+^ and CD4^+^ T cells on account of decreased MHC-I and II, and CD80. Generation of anti-tumor Th1 responses within elderly tumors may be further compromised due to reduced CD4^+^ T cells and CD11b^+^CD8α^−^CD4^+^ cDCs whose main role is activation of Th1 responses (84, 85). A reduction in the ability of elderly CD11c^+^ cells to stimulate anti-tumor effector T cells is consistent with other studies showing that elderly bone marrow-derived DCs are less efficient in stimulating tumor antigen-specific CD8^+^ and CD4^+^ T cells and mediating tumor regression (6, 7), and that administration of DC vaccines to elderly mice results in weak cytotoxic CD8^+^ T cell activity and does not slow tumor growth (8, 9). In addition, elderly tumor-associated CD11c^+^ cells may be further skewed toward suppressive function due to increased IL-10 and TNF-α. Production of IL-10 by DCs can lead to T cell inhibition and induction of Tregs (86, 87), and TNF has recently been shown to promote CD4^+^ T cell exhaustion in chronic viral infection (88). Changes in elderly tumor-associated CD11c^+^ cells suggest that the outcome of cross-talk between CD11c^+^ cells and T cells in tumors is likely to be skewed toward generation of suppressive T cells. This is supported by this study showing that elderly tumor-associated CD8^+^ and CD4^+^ T cells had reduced IFN-γ, implying reduced effector activity. Furthermore, elderly tumor-associated CD8^+^ CTLs also demonstrated compromised capacity to mediate tumor antigen-specific cell lysis and killing. Our observations agree with other studies showing that T cells in elderly tumor-bearing hosts are dysfunctional due to impaired cytotoxic activity and reduced production of IFN-γ and IL-2 (10–18). Additionally, our data suggest that elderly tumor-infiltrating CD8^+^ T cells are skewed toward suppressive function, due to increased CD73, suggesting increased potential to generate adenosine. Our parallel studies have also shown that elderly mesothelioma tumors contain increased tumor-associated macrophages (Duong et al. under review) which represent another layer of immune suppression. Thus, the elderly tumor microenvironment represents another site of increased immune suppression, which may disable local anti-tumor immune responses contributing to faster mesothelioma growth.

The increased regulatory status of elderly DCs and T cells may not only affect tumor progression, but also the efficacy of anti-tumor immunotherapies, which is supported by our data showing that IL-2/CD40 immunotherapy is less effective in inducing mesothelioma regression in elderly mice. IL-2/CD40 slowed tumor growth in both age groups (completely in young, partially in elderly) and this may be explained by our observations that IL-2/CD40 induced several common effects on tumor-infiltrating CD11c^+^ cells, CD8^+^ T cells, and CD4^+^ T cells from both age groups. Elevated IFN-γ expression by CD11c^+^ cells, and increased CD25 expression by CD8^+^ T cells suggests these cells are more activated with increased effector function and contribute to IL-2/CD40-induced slowing of tumor growth. This is supported by studies from our laboratory showing that CD8^+^ T cells are important anti-tumor effector cells during IL-2/CD40 treatment (26, 29), and other studies showing that DCs, CD8^+^ T cells and IFN-γ are required for IL-2/CD40-mediated tumor regression (89–92). Additionally, reduced CD25, CD73, and CTLA-4 on tumor-infiltrating CD4^+^ T cells in both age groups suggest reduced Treg-mediated suppression within IL-2/CD40-treated tumors. This is supported by other studies showing that IL-2/CD40 alleviates Treg suppression in murine tumors (89, 93). Importantly, IL-2/CD40 also reduced expression of several regulatory markers on young and elderly TDLN CD11c^+^ cells (CD39, CD73, TGF-β, and IL-10), suggesting alleviation of adenosine, TGF-β and IL-10-mediated suppression in TDLNs, which may create a more permissive environment for generation of IL-2/CD40-induced effector T cell responses. Collectively, these changes may contribute to the IL-2/CD40-induced slowing of tumor growth observed in both age groups.

Nonetheless, IL-2/CD40 was less effective in elderly tumor-bearing mice, which could be attributed to an exacerbated suppressive environment in elderly TDLNs. Although MHC-I and II increased on TDLN CD11c^+^ cells in elderly IL-2/CD40-treated mice, suggesting increased antigen presentation to T cells, this is likely to be accompanied by negative co-stimulatory signals, due to concomitant reductions in CD40 and CD80, and the observation that IL-2/CD40 did not overcome age-related increases in suppressive CD73 and TGF-β on elderly CD11c^+^ cells. Moreover, elderly TDLN CD8^+^ and CD4^+^ T cells may be more suppressive/exhausted and more responsive to negative signals from antigen-presenting cells, as age-related increases in CTLA-4, ICOS, TGF-β, A2AR, CD39, LAG-3, and/or PD-1 were further enhanced during IL-2/CD40 treatment. Further evidence for reduced generation of effector T cells in TDLNs of IL-2/CD40-treated elderly mice comes from the observation that elderly TDLN CD8^+^ T cells failed to up-regulate perforin and antigen-specific cytolytic effector function in response to IL-2/CD40, unlike their younger counterparts; this shows that anti-tumor cytotoxic effector T cell responses are compromised in the elderly. As previous studies from our laboratory have shown that CD8^+^ T cells and perforin-associated effector function are important for IL-2/CD40-mediated tumor regression in young mice (26), this likely contributes to poorer tumor responses by elderly mice to IL-2/CD40.

IL-2/CD40 alleviated suppression in young, but not elderly tumors, which also accounts for the better responses of young mice to IL-2/CD40 immunotherapy. Specifically, IL-2/CD40 reduced expression of several regulatory molecules (CD39, A2AR, A2BR, PD-L1, ICOS, and/or IL-10) on young tumor-infiltrating CD11c^+^ cells and CD4^+^ T cells, suggesting reduced suppressive activity. In contrast, CD11c^+^ cells and T cells infiltrating elderly IL-2/CD40-treated tumors were characterized by an increased regulatory and reduced effector status. Elderly CD11c^+^ cells displayed reduced CD40 expression, suggesting a reduced ability to license T cells (94, 95). This was associated with: (i) increased A2BR, implying an increased capacity of CD11c^+^ cells to respond to adenosine, leading to induction of tolerogenic/inhibitory DCs (96–98), and (ii) increased TGF-β, via which DCs suppress effector T cells and promote Tregs (99, 100); the latter is supported by elevations in Treg proportions and CTLA-4 expression on CD4^+^ T cells in elderly IL-2/CD40-treated tumors. Moreover, elderly tumor-associated CD8^+^ T cells demonstrated decreased perforin and IFN-γ implying reduced effector function; this is supported by our observation that elderly tumor-associated CTLs failed to up-regulate lytic function in response to IL-2/CD40, and represents another mechanism which reduces the efficacy of IL-2/CD40-mediated tumor regression in the elderly (26, 89, 90, 92). As the age-related differences in TDLN and tumor-infiltrating CD11c^+^ cells and T cells reported in this study were observed after one-third of the usual IL-2/CD40 schedule, further studies are required to examine CD11c^+^ cells/DCs and T cells after a complete schedule of IL-2/CD40.

Collectively, our data suggests that checkpoint blockade of inhibitory molecules may improve responses to IL-2/CD40 immunotherapy in elderly hosts with mesothelioma. In particular, blockade of multiple inhibitory molecules (CD39, CD73, A2A, and A2B receptors, PD-L1, PD-1, CTLA-4, LAG-3, ICOS, and TGF-β) will help reduce the likelihood of negative cross-talk during DC/T cell interactions in elderly TDLNs and tumors, thereby permitting activation of anti-tumor effector T cells. A few recent studies have reported promising results for elderly melanoma, non-small cell lung cancer and renal cancer patients treated with CTLA-4 or PD-1 blockade, with elderly patients demonstrating similar survival benefits and ability to tolerate treatments, compared to younger patients (101–104), this supports further investigation of checkpoint blockade as a therapeutic avenue for elderly mesothelioma patients.

In conclusion, our study has shown that aging programs a suppressive immune environment in healthy elderly LNs, characterized by increased suppressive pDCs, and elevated expression of several regulatory markers on CD11c^+^ cells and T cells. This regulatory environment is maintained in TDLNs of elderly mesothelioma-bearing mice, and is accompanied by increased suppression in the elderly tumor microenvironment. Thus, there is a greater summation of negative signals during elderly DC/T cell interactions, leading to effector T cell suppression and/or generation of Tregs, resulting in reduced anti-mesothelioma immune responses, that altogether contribute to faster tumor growth. Furthermore, the age-related suppressive status of elderly TDLN CD11c^+^ cells and T cells was exacerbated during IL-2/CD40 immunotherapy, which may have contributed to its reduced efficacy in elderly mice with mesothelioma. These data suggest that combining IL-2/CD40 with checkpoint blockade targeting multiple regulatory molecules could improve the efficacy of anti-cancer therapies in elderly hosts with mesothelioma.

## Ethics statement

This study was carried out in accordance with the recommendations of the Australian Code of Practice for the care and use of animals for scientific purposes. The protocol was approved by the Curtin University Animal Ethics Committee (approval number AEC-2012-21).

## Data availability

All datasets generated and analyzed for this study are included in the manuscript and the supplementary files.

## Author contributions

DN and CJ conceptualized and designed the project and experiments, were responsible for management and co-ordination of research activity and acquired financial support for the project. DN, CJ, and CM provided supervision for research activity planning and study execution. CJ and JG performed the experiments. JG, CJ, and DN analyzed the data. All authors contributed to writing, reviewing and editing the manuscript.

### Conflict of interest statement

DN acts as a non-salaried Chief Scientific Officer for Selvax. Selvax did not fund or contribute to this study in any way. The remaining authors declare that the research was conducted in the absence of any commercial or financial relationships that could be construed as a potential conflict of interest.
