# Supplementary figures and images for: The Regulatory Status Adopted by Lymph Node Dendritic Cells and T Cells During Healthy Aging Is Maintained During Cancer and May Contribute to Reduced Responses to Immunotherapy
Source: Front Med (Lausanne). 2018 Nov 30;5:337. doi: 10.3389/fmed.2018.00337 (PMC6287204; doi:10.3389/fmed.2018.00337)

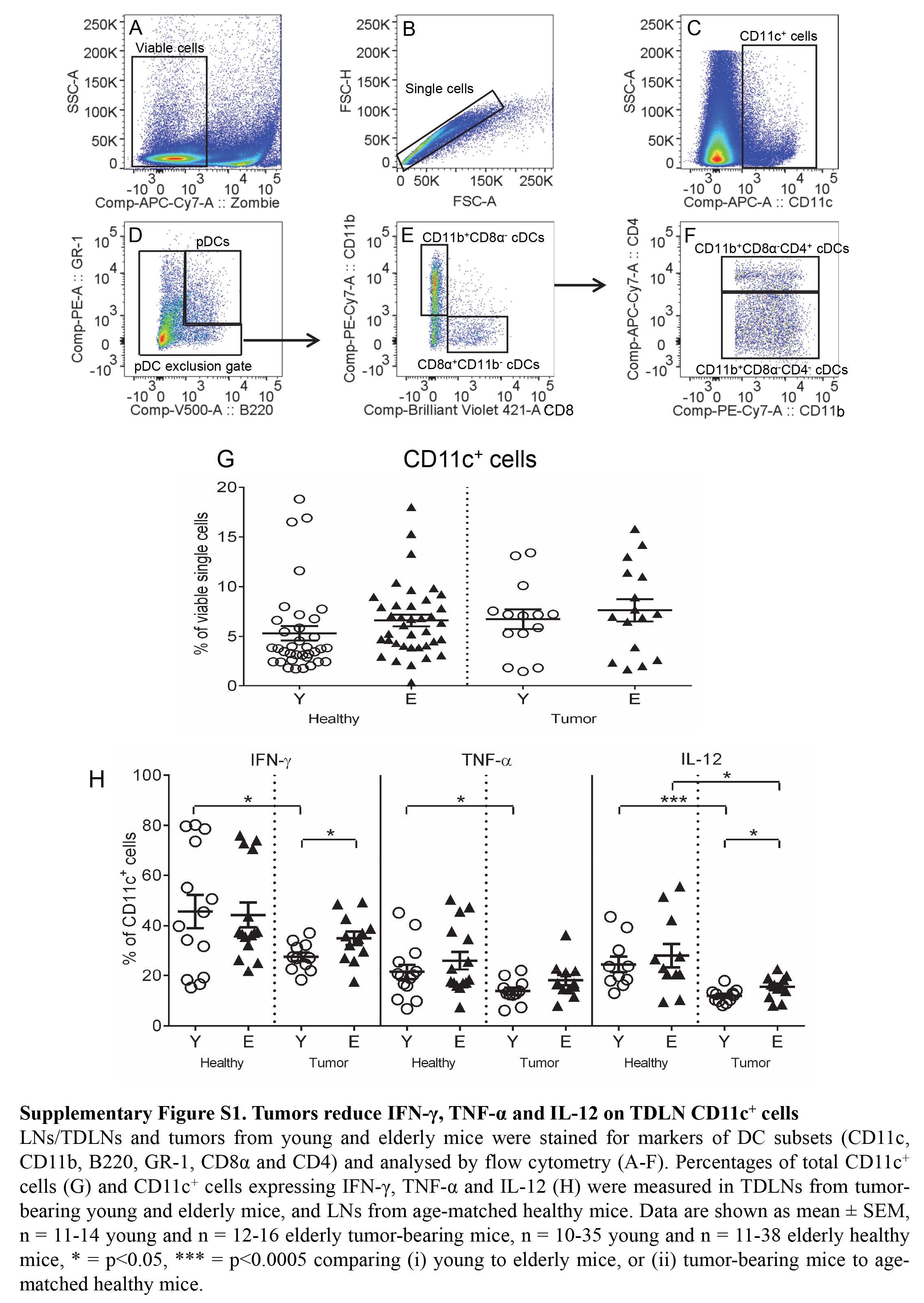

Supplement: Supplementary file 1 [file Image_1.TIF]

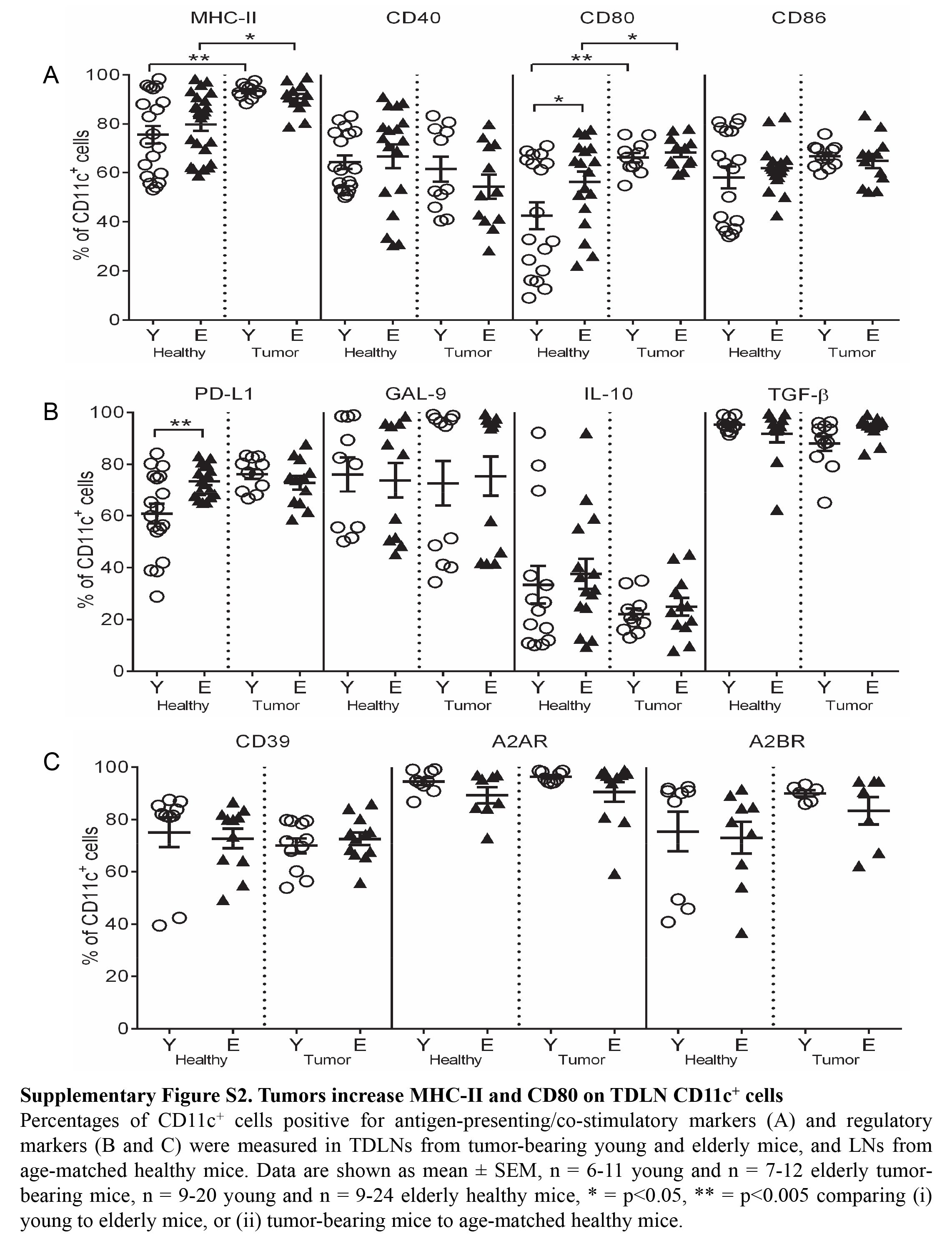

Supplement: Supplementary file 2 [file Image_2.TIF]

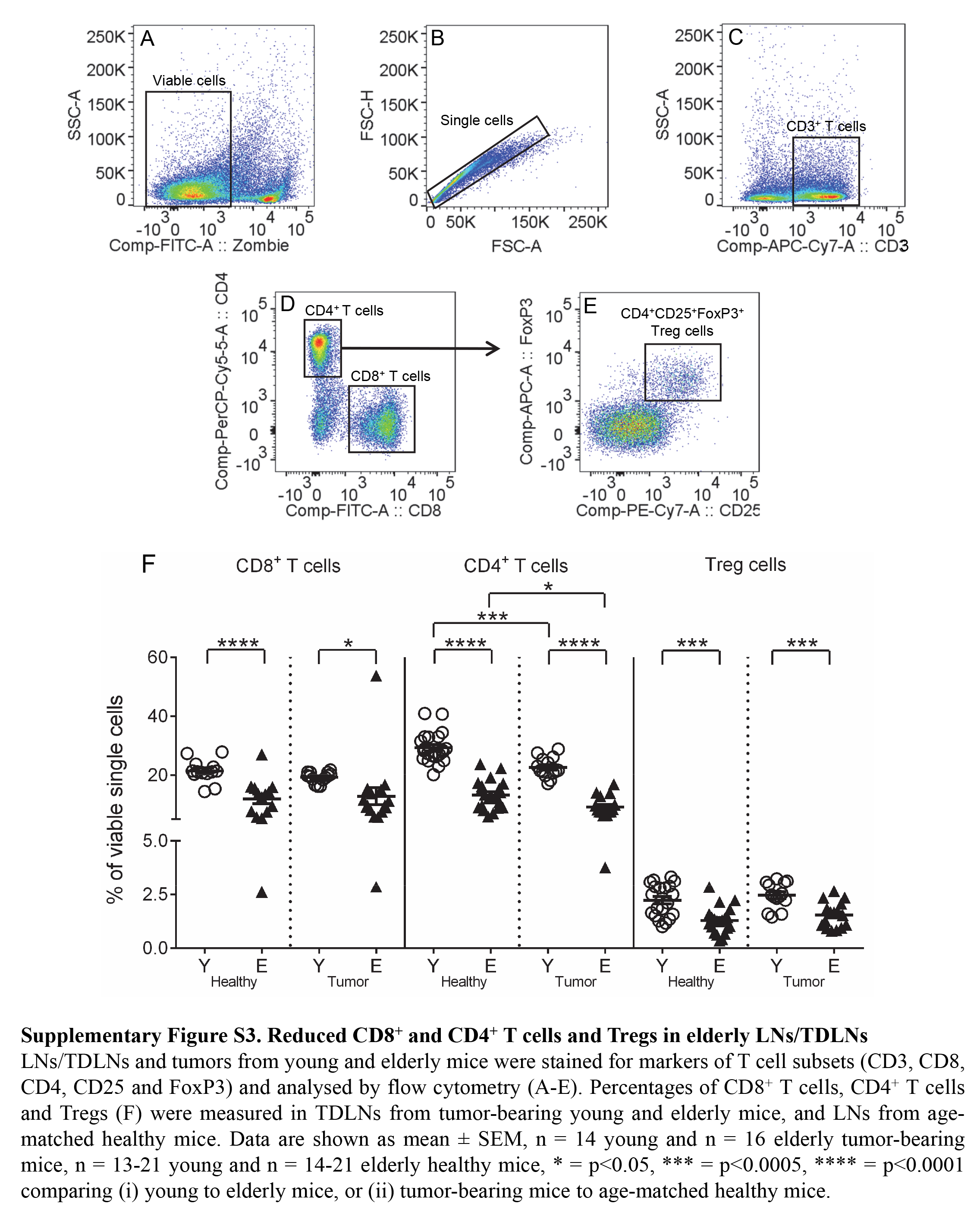

Supplement: Supplementary file 3 [file Image_3.TIF]

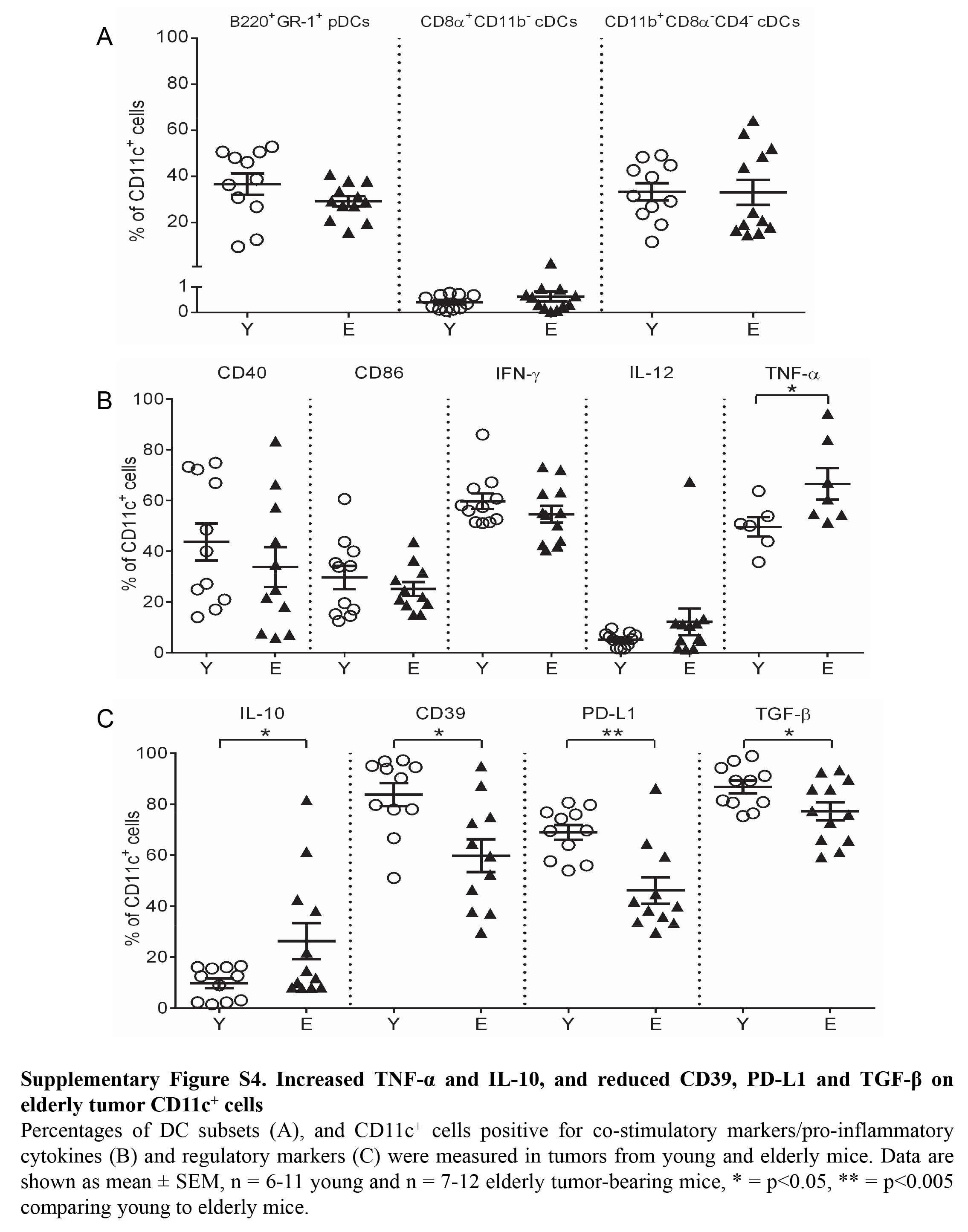

Supplement: Supplementary file 4 [file Image_4.TIF]

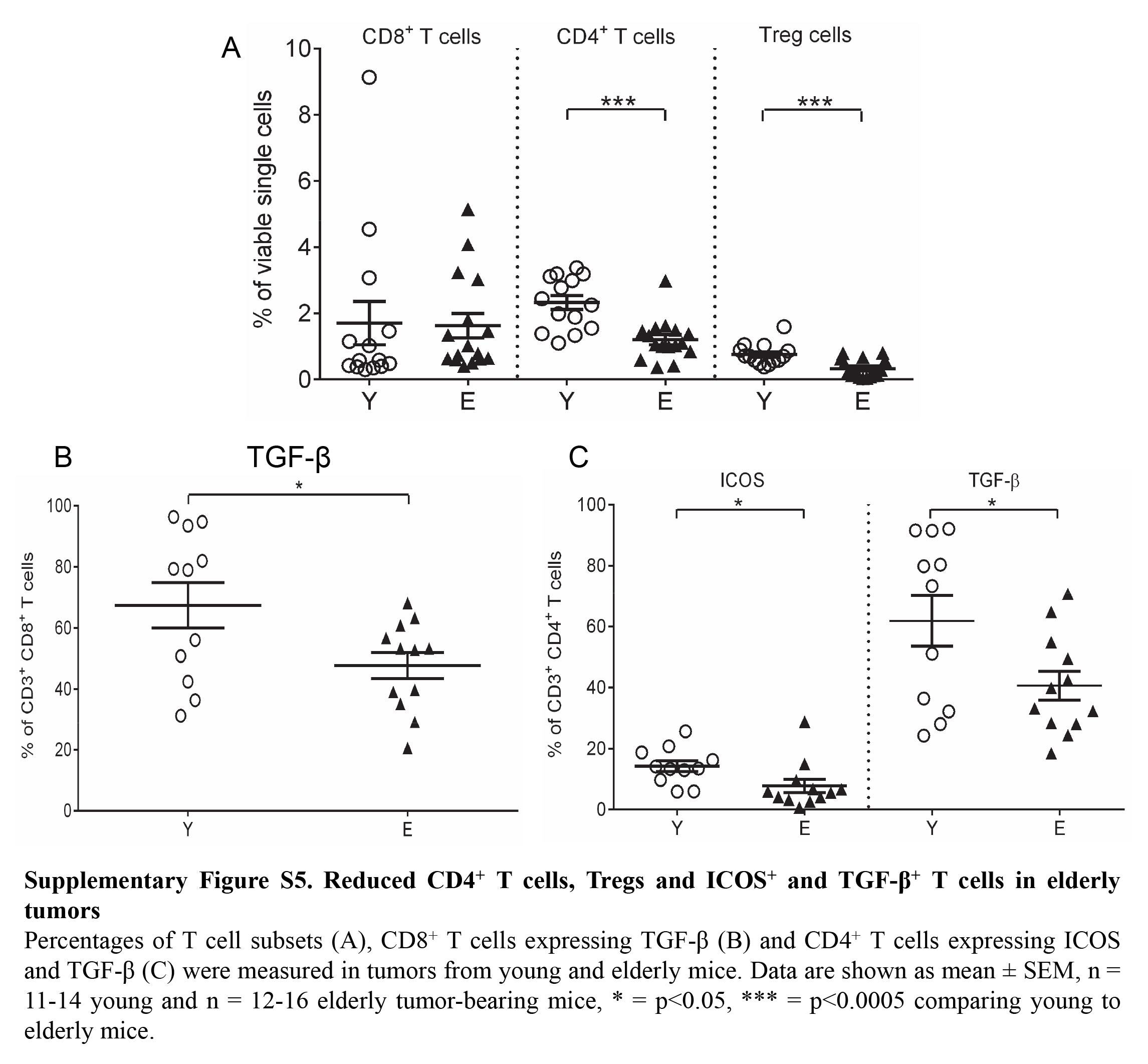

Supplement: Supplementary file 5 [file Image_5.TIF]

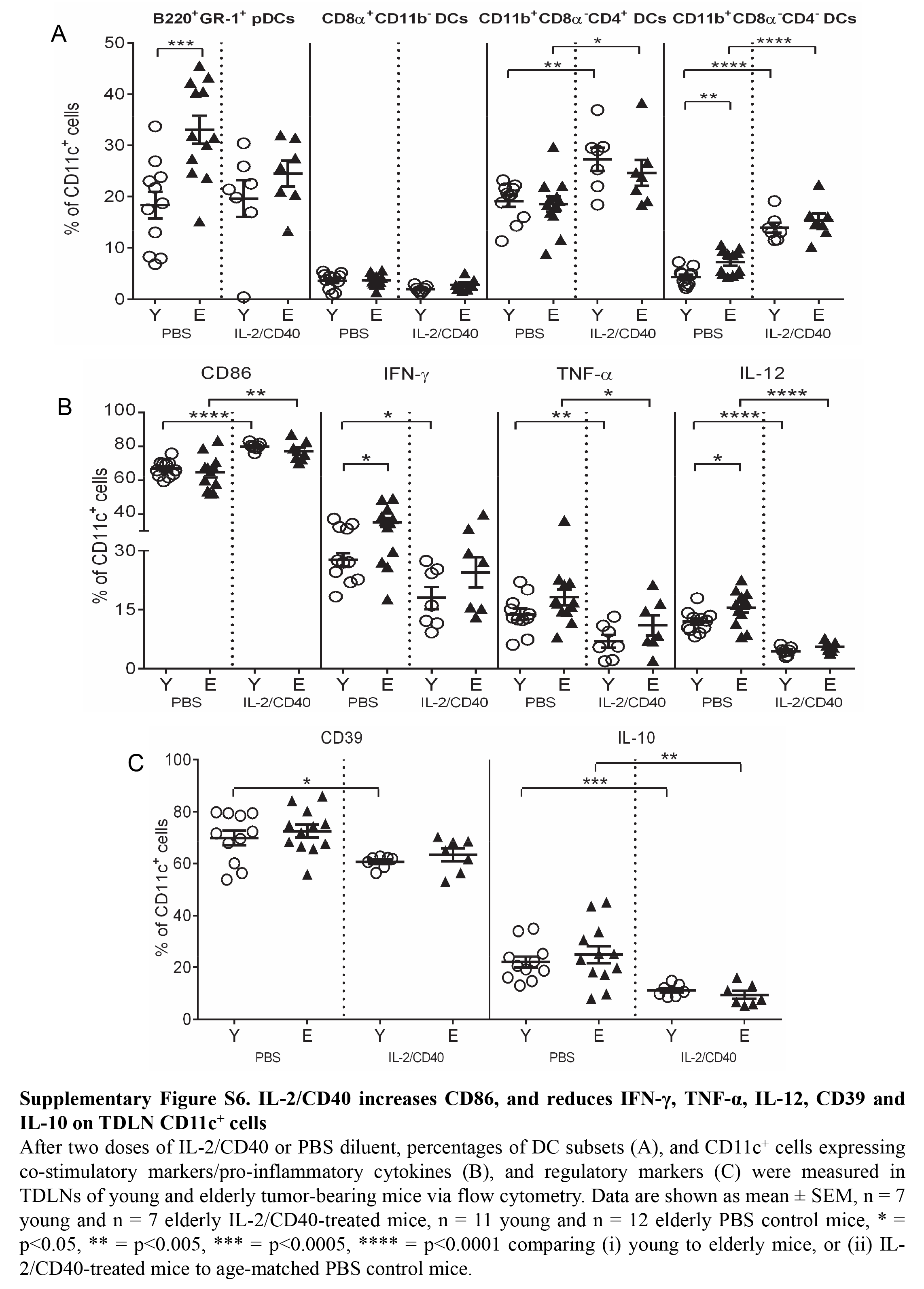

Supplement: Supplementary file 6 [file Image_6.TIF]

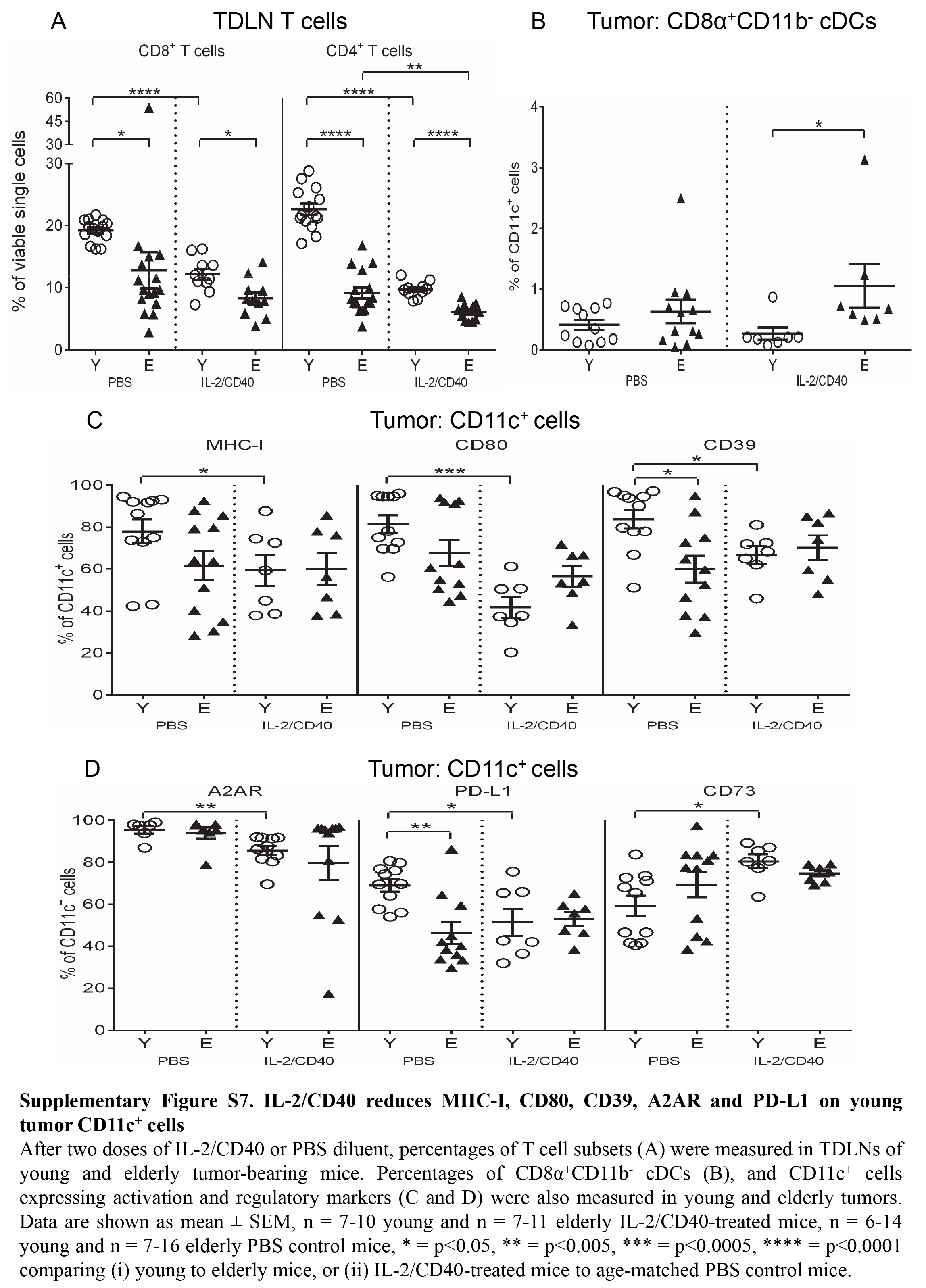

Supplement: Supplementary file 7 [file Image_7.TIF]

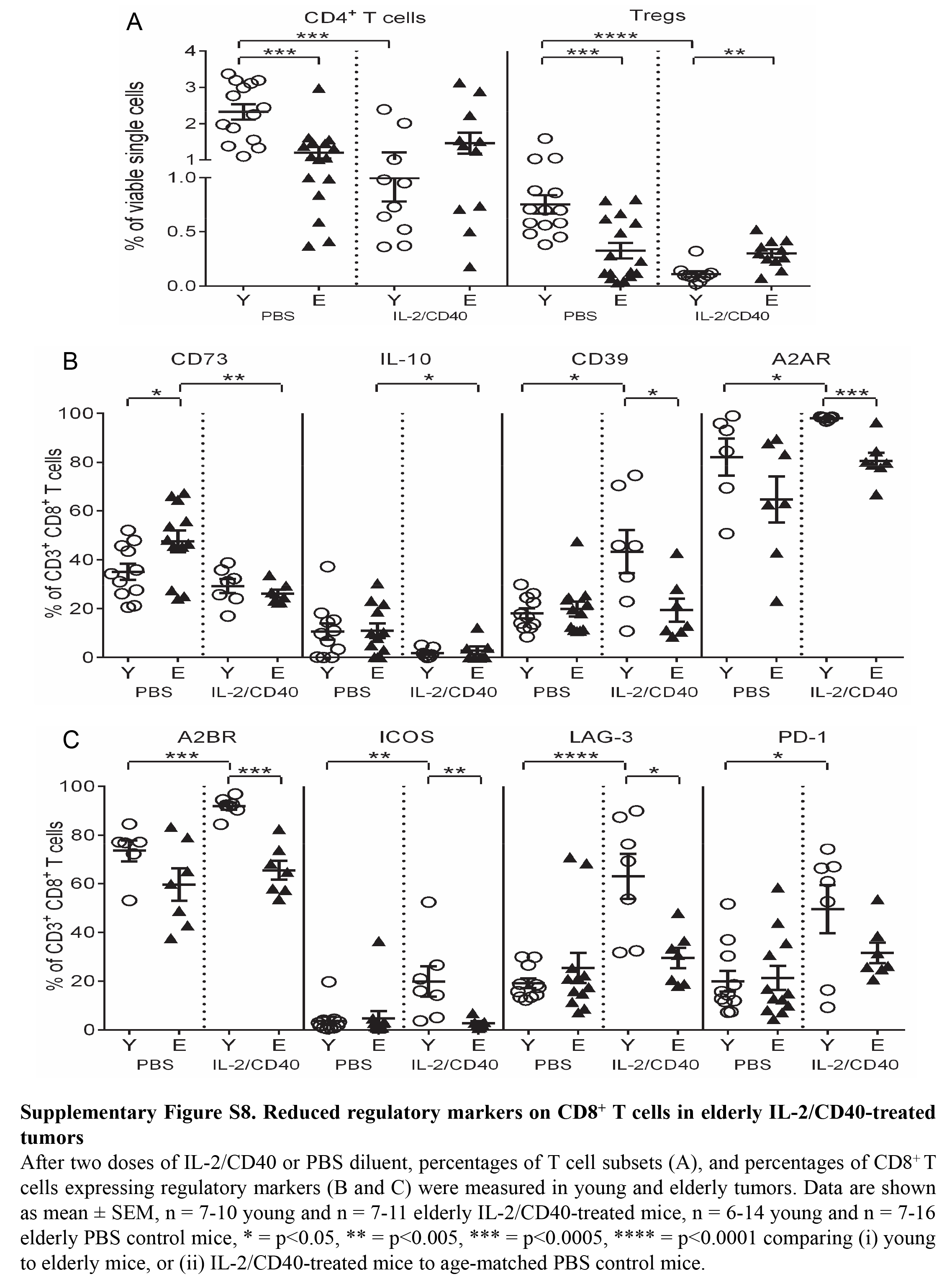

Supplement: Supplementary file 8 [file Image_8.TIF]
